# Consensus In Silico Pharmacodynamic Screening of Twelve Fabaceae Isoflavones Targeting Glycation, Oxidative Stress, Antidiabetic Activity, and NF-κB-Related Inflammation

**DOI:** 10.3390/ph19091353

**Published:** 2026-08-26

**Authors:** Saied A. Aboushanab, Denis A. Oberiukhtin, Irina G. Danilova, Pavel M. Vassiliev, Ksenia V. Sokolova, Kewei Chen, Elena G. Kovaleva, Alicia C. Mondragon, Ekaterina I. Demicheva, Jose M. Miranda

**Affiliations:** 1 Institute of Chemical Engineering, Ural Federal University Named After the First President of Russia B. N. Yeltsin, Mira 19, 620002 Yekaterinburg, Russia; e.g.kovaleva@urfu.ru (E.G.K.); fimby@mail.ru (E.I.D.); 2 Institute of Immunology and Physiology, Ural Branch of the Russian Academy of Science, 106 Pervomaiskaya Street, 620049 Yekaterinburg, Russia; oberuhtindenis@gmail.com (D.A.O.); i.g.danilova@urfu.ru (I.G.D.); ksenia.sokolova@urfu.ru (K.V.S.); 3 Institute of Natural Science and Mathematics, Ural Federal University Named After the First President of Russia B. N. Yeltsin, Mira 19, 620002 Yekaterinburg, Russia; 4 Department of Pharmacology and Bioinformatics, Volgograd State Medical University, 1 Pavshikh Bortsov Sq., 400131 Volgograd, Russia; pvassiliev@mail.ru; 5 College of Food Science, Southwest University, No. 2, Tiansheng Road, Beibei, Chongqing 400715, China; chenkewei@swu.edu.cn; 6 Department of Analytical Chemistry, Nutrition and Food Science, Institute for Research in Global Health and Sustainable Development, iTERRA, School of Veterinary Sciences, University of Santiago de Compostela, 27002 Lugo, Spain; alicia.mondragon@usc.es

**Keywords:** type 2 diabetes mellitus, isoflavones, Fabaceae, molecular docking, NF-κB, glycation, antioxidant activity, PASS, in silico screening

## Abstract

**Background/Objectives:** Type 2 diabetes mellitus (T2DM) is a multifactorial metabolic disorder characterized by chronic hyperglycemia, oxidative stress, protein glycation, low-grade inflammation, and dysregulation of signaling pathways, including those involving nuclear factor kappa B (NF-κB). Isoflavones from Fabaceae species exhibit diverse biological activities and may represent multifunctional candidates for addressing the interconnected mechanisms underlying T2DM. This study performed a consensus in silico pharmacodynamic screening of twelve representative Fabaceae isoflavones to prioritize compounds with predicted antiglycating, deglycating, antidiabetic, antioxidant, and NF-κB-related anti-inflammatory activities. **Methods:** Twelve representative Fabaceae isoflavones were evaluated using a multimethod computational workflow. Antiglycating and deglycating activities were predicted using IT Microcosm, antidiabetic and hypoglycemic activities were assessed using Prediction of Activity Spectra for Substances (PASS), antioxidant potential was evaluated using similarity-based prediction and quantum-chemical calculations, and NF-κB-related anti-inflammatory activity was investigated using similarity-based prediction combined with molecular docking. Molecular docking was applied specifically to the NF-κB-related anti-inflammatory analysis and did not contribute to the hypoglycemic/antidiabetic prediction. The individual predictions were integrated using a consensus scoring strategy to prioritize compounds across multiple pharmacodynamic domains. **Results:** The investigated isoflavones demonstrated moderate but broad predicted multifunctional activities. Malonylgenistin, biochanin A, and formononetin showed the most favorable predicted antiglycating profiles. Genistin, acetyldaidzin, acetylglycitin, and puerarin ranked highest in terms of predicted antidiabetic activity, whereas genistein exhibited the strongest predicted antioxidant profile. Daidzin and genistin received the highest integrated NF-κB-related computational rankings, although these predictions apply to intact glycosides rather than their expected in vivo hydrolysis products. **Conclusions:** This study presents a consensus computational framework for the comparative prioritization of Fabaceae isoflavones based on their predicted multifunctional pharmacodynamic profiles. The results provide a rational basis for selecting candidate compounds for future biochemical, cell-based, pharmacokinetic, and in vivo investigations, although it is emphasized that the present findings are based exclusively on computational predictions and require experimental validation.

## 1. Introduction

Type 2 diabetes mellitus (T2DM) is a complex metabolic disorder in which persistent hyperglycemia is accompanied by impaired insulin signaling, oxidative imbalance, chronic low-grade inflammation, and the progressive accumulation of advanced glycation end products (AGEs) [1,2,3]. These pathological events do not occur separately; instead, they form an interconnected network in which hyperglycemia accelerates nonenzymatic protein glycation and reactive oxygen species generation, whereas AGEs and their signaling and inflammatory mediators further contribute to endothelial dysfunction, β-cell impairment, insulin resistance, and tissue injury [4,5]. Consequently, the development of pharmacologically relevant compounds capable of addressing several diabetes-associated mechanisms in parallel remains important in medicinal chemistry, pharmacognosy, and natural-product-based drug discovery [6,7].

Plant-derived polyphenols have received considerable attention in this context because they frequently present combinations of antioxidant, anti-inflammatory, and metabolism-modulating properties [7,8]. Among them, isoflavones constitute a structurally distinct subclass of flavonoids occurring mainly in plants of the Fabaceae family. Compounds such as daidzein, genistein, puerarin, formononetin, biochanin A, glycitein, and their glycosylated or acylated derivatives are characteristic constituents of several Fabaceae sources, including *Pueraria lobata*, *Trifolium pratense*, and soybean-derived materials [9,10,11]. Previous studies have indicated that these molecules may influence oxidative stress, inflammatory signaling, glucose metabolism, and glycation-related cellular damage, although their activity profiles differ depending on the chemical structure, substitution pattern, and biological endpoint [9,10,11].

Botanical Fabaceae extracts usually consist of multiple structurally related isoflavones, including aglycones, O-glycosides, C-glycosides, acylated glycosides, and methoxylated derivatives. Therefore, their biological effects may result from the combined contribution of several constituents rather than from a single dominant compound. Although individual isoflavones have been investigated in relation to oxidative stress, inflammation, glucose metabolism, and AGE-related cellular injury, these data remain dispersed across different compounds, endpoints, and experimental or computational models [7,12,13]. Consequently, comparative in silico prioritization of individual isoflavones provides a useful step toward connecting the phytochemical composition of Fabaceae extracts with mechanism-oriented biological validation.

Despite the availability of numerous studies on individual isoflavones, their comparative multifunctional relevance to T2DM-associated mechanisms remains insufficiently systematized. Most previous investigations have focused mainly on one compound, one biological target, or one pharmacological effect [9,13]. In contrast, the pathogenesis of T2DM involves the convergence of several mutually reinforcing processes, including glycation, oxidative stress, inflammatory activation, and impaired glucose regulation [7,14]. This creates the need for an integrated evaluation strategy that moves beyond single-endpoint screening and considers compounds with balanced activity across several relevant pharmacodynamic domains.

Consensus in silico screening represents an appropriate approach for this purpose because it integrates structurally based, probability-based, similarity-based, quantum-chemical, and docking-derived predictions into a unified prioritization framework [15]. Such an approach can reduce the dependence on a single computational method and facilitate the identification of compounds that warrant subsequent experimental testing. In the present study, twelve representative Fabaceae isoflavones, such as daidzein, glycitein, genistein, puerarin, daidzin, acetyldaidzin, glycitin, acetylglycitin, genistin, malonylgenistin, biochanin A, and formononetin, were selected for comparative computational analysis. This panel includes aglycones, C-glycosides, O-glycosides, acylated glycosides, and methoxylated aglycones, thereby allowing the assessment of structurally related molecules with distinct physicochemical and pharmacodynamic features. To our knowledge, no previous study has performed a consensus comparison of structurally related Fabaceae isoflavones across multiple diabetes-related pharmacodynamic domains by integrating similarity-based prediction, quantum-chemical modeling, molecular docking, and consensus scoring within a single prioritization framework.

The aim of this work was to perform a consensus in silico screening of twelve representative Fabaceae isoflavones to compare their predicted antiglycating, deglycating, antidiabetic, antioxidant, and NF-κB-related pharmacodynamic profiles within a unified computational framework. Rather than evaluating isolated compounds or individual biological endpoints, the study was designed to generate an integrated comparative pharmacodynamic profile, identify domain-specific priority candidates, and establish a consensus-based prioritization strategy for subsequent biochemical, cell-based, absorption, distribution, metabolism, excretion and toxicity (ADMET), and in vivo validation in models relevant to T2DM and its complications. We hypothesized that structurally related Fabaceae isoflavones would exhibit distinct computational profiles across diabetes-related pharmacodynamic domains and that the consensus integration of complementary computational approaches would provide a more robust and informative prioritization than any individual predictive method alone.

## 2. Results

### 2.1. Overview of In Silico Screening Outputs

The consensus in silico workflow generated a comparative pharmacodynamic profile for twelve structurally related Fabaceae isoflavones. The evaluated panel includes aglycones, C-glycosides, O-glycosides, acylated glycosides, and methoxylated aglycones. Each compound was assessed across five diabetes-related activity domains: antiglycating activity, deglycating activity, hypoglycemic/antidiabetic potential, antioxidant potential, and NF-κB-related anti-inflammatory potential.

The computational outputs consisted of categorical predictions, similarity- and probability-based indices, quantum-chemical thermodynamic parameters, docking-derived affinity indices (generated exclusively for the NF-κB-related domain), and final consensus classifications. For comparative interpretation, the outputs were converted into standardized activity levels: high, moderate, or low. When the computational methods produced partially divergent outputs, the final category was marked with a question mark, indicating reduced confidence at the assigned level.

Across the integrated screening profile, none of the twelve isoflavones were classified as inactive in the overall multifunctional assessment. This result was not unexpected because all the selected compounds share an isoflavone/chromone polyphenolic scaffold, which is known to provide a baseline capacity for broad pharmacological interactions, including antioxidant and signaling-related effects. Therefore, the absence of inactive compounds should be interpreted as a scaffold-level baseline property rather than as the main discriminating outcome of the screening. The most informative result was the domain-specific differentiation among structurally related compounds. High predicted antiglycating activity was observed for malonylgenistin, biochanin A, and formononetin. High predicted hypoglycemic/antidiabetic activity was observed for acetylglycitin, puerarin, genistin, and acetyldaidzin. The antioxidant profile of genistein was the most consistent, whereas those of daidzein, glycitein, puerarin, and formononetin were assigned to the high-antioxidant category with reduced confidence. In the NF-κB-related assessment, daidzin and genistin were associated with the highest final consensus classifications.

**Figure 1:**
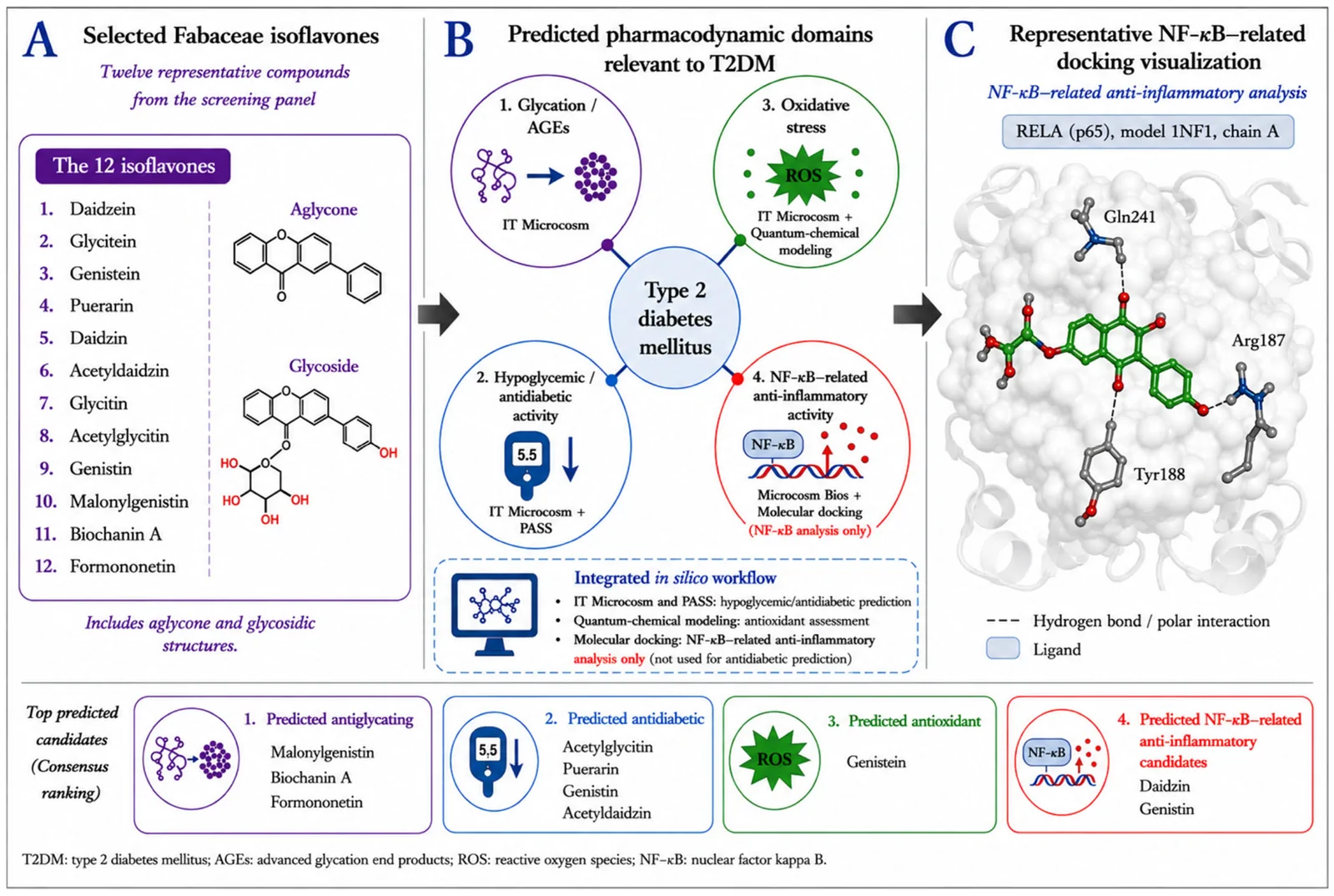
Integrated in silico screening workflow and mechanistic prioritization of representative Fabaceae isoflavones. (**A**) Representative selected isoflavones from the twelve-compound panel, including aglycone and glycosidic structures. (**B**) Predicted pharmacodynamic domains relevant to type 2 diabetes mellitus, including glycation/advanced glycation end product formation (purple), oxidative stress (green), hypoglycemic/antidiabetic activity (blue), and NF-κB-related inflammation (red). The consensus computational workflow included similarity-based prediction, quantum-chemical modeling, and molecular docking (exclusively for NF-κB-related anti-inflammatory prediction). (**C**) Representative schematic visualization of the docking grid and ligand placement within an NF-κB-related binding site. The lower panel summarizes the main domain-specific priority candidates identified by consensus screening.

These results demonstrate that the studied isoflavones did not belong to a uniform pharmacodynamic group. Instead, the screening revealed domain-specific activity patterns that enabled the prioritization of individual compounds for different diabetes-related mechanisms. The overall computational workflow and the main activity-specific priority candidates are summarized in Figure 1.

### 2.2. Predicted Antiglycating Activity

The predicted antiglycating activity of the twelve isoflavones was assessed using the information technology (IT) Microcosm consensus strategy. The final classification integrated conservative, normal, and risk-based prediction outputs (Table 1).

Three compounds were classified as having “high?” predicted antiglycating activity: malonylgenistin, biochanin A, and formononetin. Malonylgenistin received a high final consensus classification because the risk-based strategy indicated high activity, while the conservative and normal strategies indicated moderate activity. Biochanin A and formononetin were classified as high by the conservative strategy, although the normal and risk-based strategies produced nonconclusive outputs.

The remaining nine compounds were classified as “moderate” or “moderate?”. For glycitein, daidzin, acetyldaidzin, glycitin, acetylglycitin, and genistin, moderate classification was supported by all three prediction strategies. Daidzein and puerarin were classified as moderate mainly through the conservative strategy, whereas genistein was classified as moderate through the risk-based strategy. Overall, the antiglycation prediction identified malonylgenistin, biochanin A, and formononetin as the main priority candidates in this activity domain.

### 2.3. Predicted Deglycating Activity

The predicted deglycating activity of the selected isoflavones was assessed using the IT Microcosm consensus strategy. The final classification was derived from conservative, normal, and risk-based prediction outputs (Table 2).

No compound was classified as having high predicted deglycating activity. Six compounds were assigned to the moderate category: acetyldaidzin, acetylglycitin, biochanin A, formononetin, glycitin, and malonylgenistin. All these showed concordant moderate classifications across all three strategies.

Daidzein, daidzin, genistein, genistin, and puerarin were classified into moderate categories with reduced confidence because of incomplete agreement between the prediction strategies. Glycitein was the only compound classified as low. Thus, the deglycating endpoint was less differentiated than the antiglycating endpoint, with most compounds showing moderate predicted activity and no compound reaching a high classification.

### 2.4. Predicted Hypoglycemic and Antidiabetic Activity

The predicted hypoglycemic and antidiabetic potential of the twelve isoflavones was evaluated using Prediction of Activity Spectra for Substances (PASS) and IT Microcosm-derived activity indices. PASS provided probability-based outputs for hypoglycemic and antidiabetic activity, whereas the final consensus index integrated PASS-derived and IT Microcosm-derived estimates. No molecular docking-derived parameters were used in the hypoglycemic/antidiabetic prediction or consensus ranking. The results are presented in Table 3 and Table 4. In the PASS-based analysis, puerarin had the highest integrated PASS-derived index, with a consensus value of 3.0, followed by genistin, daidzin, acetyldaidzin, and acetylglycitin, each with a PASS-derived consensus index of 2.5. Glycitin and malonylgenistin also showed favorable PASS-derived profiles, with a consensus index of 2.0. Lower PASS-derived indices were observed for biochanin A, genistein, formononetin, daidzein, and glycitein.

After integration with IT Microcosm-derived estimates, four compounds were classified as having high predicted antidiabetic activity: acetylglycitin, puerarin, genistin, and acetyldaidzin. Their final consensus indices ranged from 1.9 to 2.3. Acetylglycitin had the highest final consensus index (IndC = 2.3), followed by puerarin and genistin (IndC = 2.0 each) and acetyldaidzin (IndC = 1.9). Six compounds were classified as moderate: daidzin, malonylgenistin, biochanin A, glycitin, formononetin, and daidzein. Their final consensus indices ranged from 0.8 to 1.5. Two compounds, genistein and glycitein, were classified as having low predicted antidiabetic activity, with final consensus indices of 0.6 and 0.5, respectively.

### 2.5. Predicted Antioxidant Activity

The antioxidant potential of the selected isoflavones was evaluated using similarity-based prediction and quantum-chemical modeling of hydroxyl radical oxidation. The similarity-based approach generated TQL values and categorical antioxidant predictions, whereas the quantum-chemical approach provided the selected Gibbs free energy value for the most favorable modeled hydroxyl radical oxidation pathway. The integrated results are presented in Table 5. The similarity-based assessment classified genistein as the only compound with high predicted antioxidant activity. Seven compounds, such as daidzein, glycitein, puerarin, daidzin, genistin, biochanin A, and formononetin, were classified as moderate, whereas acetyldaidzin, glycitin, acetylglycitin, and malonylgenistin were classified as low by this approach. The quantum-chemical model produced a different distribution of activity categories. Daidzein, glycitein, puerarin, acetyldaidzin, malonylgenistin, and formononetin were classified as above moderate on the basis of the hydroxyl radical oxidation model. Genistein, acetylglycitin, and genistin were classified as moderate, while daidzin, glycitin, and biochanin A were classified as below moderate.

After the integration of both antioxidant prediction outputs, genistein, daidzein, glycitein, puerarin, and formononetin were classified as high, indicating potentially high antioxidant activity but with reduced confidence due to partial disagreement between the two methods. Genistin was classified as moderate, while acetyldaidzin and malonylgenistin were assigned to the moderate category with reduced confidence. Glycitin was classified as low, whereas daidzin, acetylglycitin, and biochanin A were assigned to the low category with reduced confidence because of partial divergence between the two antioxidant prediction approaches. Overall, the results of the antioxidant prediction revealed that genistein was the most consistent antioxidant candidate across the two computational approaches. Daidzein, glycitein, puerarin, and formononetin also showed favorable predicted antioxidant profiles, although their final categories were assigned with reduced confidence. The main numerical indices used for consensus classification, including antidiabetic IndC, antioxidant TQL and ΔGMin, and NF-κB-related IndMC_BioS, IndDock, and IndNF-κB, are summarized in Table S1.

### 2.6. Predicted NF-κB-Related Anti-Inflammatory Potential

The NF-κB-related anti-inflammatory potential of the selected isoflavones was evaluated using similarity-based prediction and molecular docking. The similarity-based analysis generated the IndMC_BioS index, whereas molecular docking generated binding energy values and the corresponding IndDock values. These two outputs were integrated into the final consensus index IndNF-κB, which was used to assign the final activity category. The results are presented in Table 6. The similarity-based Microcosm BioS prediction produced relatively low to moderate index values across the compound set. The highest IndMC_BioS values were observed for daidzein and formononetin, whereas the lowest values were observed for acetylglycitin, glycitein, and malonylgenistin. In contrast, the docking-based assessment predicted favorable binding profiles for all twelve compounds, with docking-derived indices ranging from 1.9 to 4.1.

The strongest docking-derived profiles were observed for daidzin and genistin, both of which had an IndDock value of 4.1. Acetyldaidzin and malonylgenistin also had relatively high docking-derived indices, with values of 3.6 and 3.1, respectively. The remaining compounds showed moderate-to-favorable docking-derived indices ranging from 1.9 to 2.4. Integration of the similarity-based and docking-derived indices placed genistin and daidzin in the highest NF-κB-related computational score tier, with IndNF-κB values of 2.1 and 2.0, respectively. These values represent relative computational rankings within the investigated compound panel and do not demonstrate functional NF-κB inhibition or anti-inflammatory activity. The other ten compounds were classified as moderate, with IndNF-κB values ranging from 0.5 to 1.4. No compound was classified as low in the final NF-κB-related consensus assessment. Overall, the NF-κB-related screening identified genistin and daidzin as the strongest candidates in this activity domain. The remaining isoflavones received moderately integrated NF-κB-related computational scores. The relatively narrow score range among these compounds indicates limited differentiation within the applied computational framework, while daidzin and genistin ranked highest. These results provide a relative ranking for follow-up testing but do not establish an indication of NF-κB pathway modulation.

**Figure 2:**
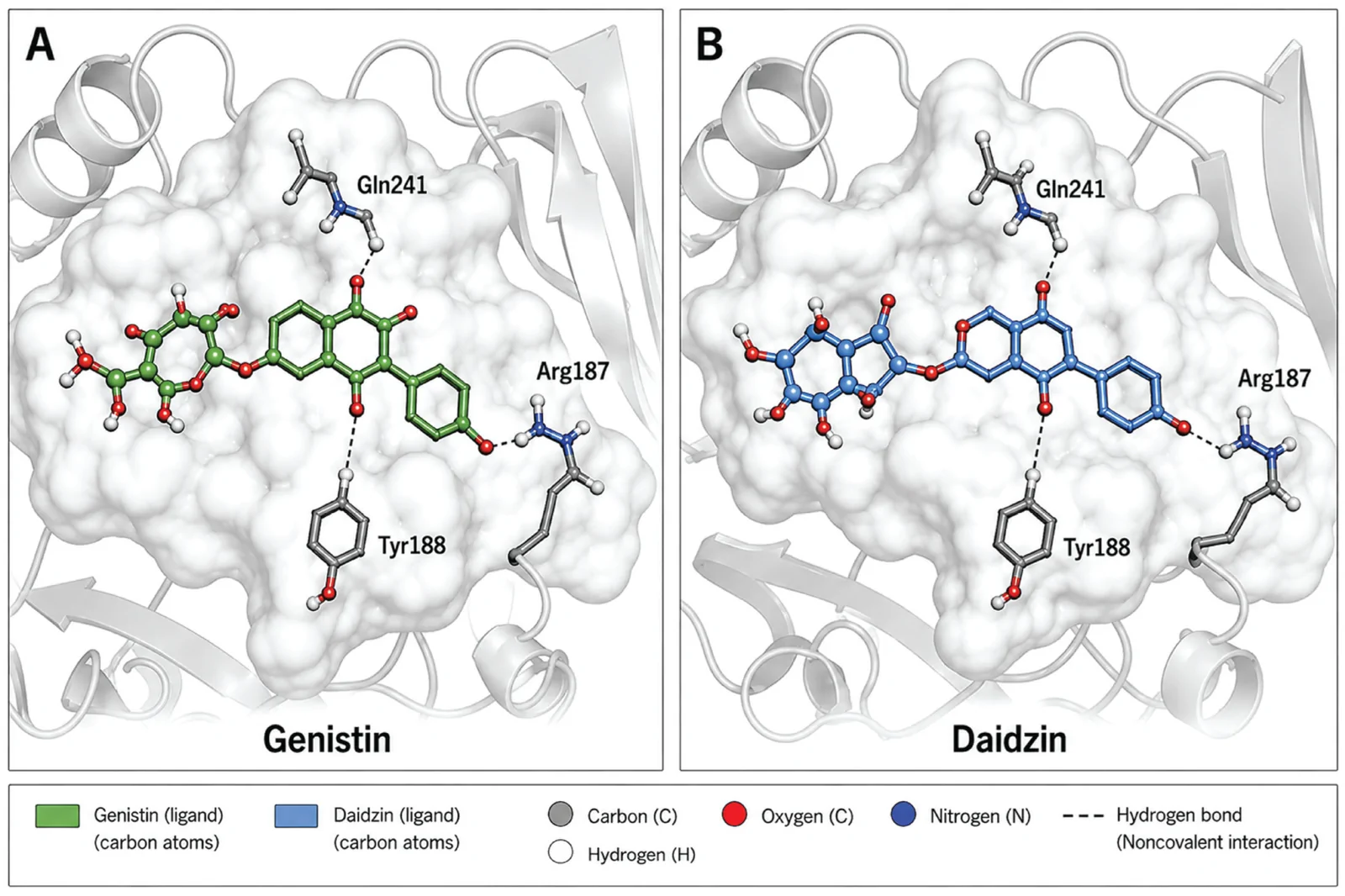
(**A**) Schematic docking visualization of genistin in the predicted NF-κB-related binding region. (**B**) Schematic docking visualization of daidzin in the predicted NF-κB-related binding region. The protein pocket is shown as a semitransparent gray surface, while the ligands are shown in gray stick representation. Genistin is shown in green and daidzin in light blue; oxygen and nitrogen atoms are shown in red and blue, respectively. Dashed black lines indicate possible noncovalent interactions that may contribute to ligand stabilization within the binding region. Genistin and daidzin were selected for visualization because they showed the highest docking-derived indices and the highest final NF-κB-related consensus classifications among the studied isoflavones.

To provide a visual interpretation of the docking-based component of the NF-κB-related screening, a representative schematic visualization was prepared for the two compounds with the highest final NF-κB-related consensus scores, namely, genistin and daidzin (Figure 2). Both compounds showed the strongest docking-derived profiles among the evaluated isoflavones, with ΔE values of −9.4 kcal/mol and IndDock values of 4.1. The predicted placement of these glycosidic isoflavones in an NF-κB-related binding site environment is shown in Figure 2, which highlights the possible formation of stabilizing noncovalent interactions.

### 2.7. Integrated Multifunctional Consensus Profile and Domain-Specific Prioritization

The activity-specific outputs from the five predicted pharmacodynamic domains (antiglycating, deglycating, hypoglycemic/antidiabetic, antioxidant, and NF-κB-related anti-inflammatory activity) were integrated to generate a comparative multifunctional profile for the twelve selected isoflavones (Table 7). All twelve compounds were classified as having an overall moderate multifunctional consensus profile, reflecting the absence of inactive compounds across the screening set and the presence of at least moderate predicted activity in several domains for each isoflavone. However, the domain-specific patterns differed considerably among compounds, allowing the prioritization of individual candidates according to their strongest predicted activities.

Malonylgenistin, biochanin A, and formononetin showed the most favorable predicted antiglycating profiles. Acetylglycitin, puerarin, genistin, and acetyldaidzin were prioritized for their predicted hypoglycemic/antidiabetic activity. Genistein, daidzein, glycitein, puerarin, and formononetin were all assigned to the high-antioxidant category with reduced confidence (High?) due to partial divergence between the similarity-based and quantum-chemical outputs, with genistein showing the most consistent profile among them. With respect to NF-κB-related anti-inflammatory potential, daidzin and genistin were the strongest predicted candidates, with no other compound reaching a high classification in this domain. No compound was classified as having high deglycating activity; this endpoint was less differentiated overall, with most compounds falling into the moderate category.

When the five domains were considered together, the combined profiles of genistin and puerarin were particularly favorable. Genistin strongly predicted antidiabetic activity and NF-κB-related activity but moderately predicted antiglycating, deglycating, and antioxidant activity. Puerarin combined high predicted antidiabetic activity with a potentially high (although reduced confidence) antioxidant profile and moderately predicted antiglycating and NF-κB-related activity. Formononetin and malonylgenistin were also notable for their high predicted antiglycating activity, and acetyldaidzin and daidzin were distinguished by strong domain-specific antidiabetic and NF-κB-related profiles, respectively. On the basis of this combined evidence, genistin, puerarin, formononetin, malonylgenistin, acetyldaidzin, and daidzin emerged as the priority compounds for further target interaction validation.

Genistin, acetyldaidzin, and daidzin are O-glycosides that are subject to extensive intestinal β-glucosidase and microbial hydrolysis, such that the aglycone (and its phase-II conjugates) rather than the intact glycoside is expected to be the dominant circulating and tissue-exposed species in vivo [16]. Their prioritization here reflects the predicted structure–target complementarity of the intact molecule and should not be read as an indicator of achievable systemic exposure or in vivo efficacy of the parent compound. Puerarin, in contrast, possesses a C-glycosidic bond that is resistant to β-glucosidase hydrolysis and shows demonstrable oral absorption in vivo [17], suggesting that its predicted profile has greater direct pharmacokinetic plausibility. Experimental follow-up for O-glycosidic candidates should therefore evaluate both the parent glycoside and its principal aglycone/metabolite (genistein, daidzein) in parallel to establish which chemical form is responsible for any confirmed activity.

Overall, the results of the integrated analysis indicate that no single compound was superior across all five pharmacodynamic domains. Instead, the most promising candidates differed according to the target activity domain, supporting a domain-specific validation strategy rather than the selection of a single universally superior isoflavone.

**Figure 3:**
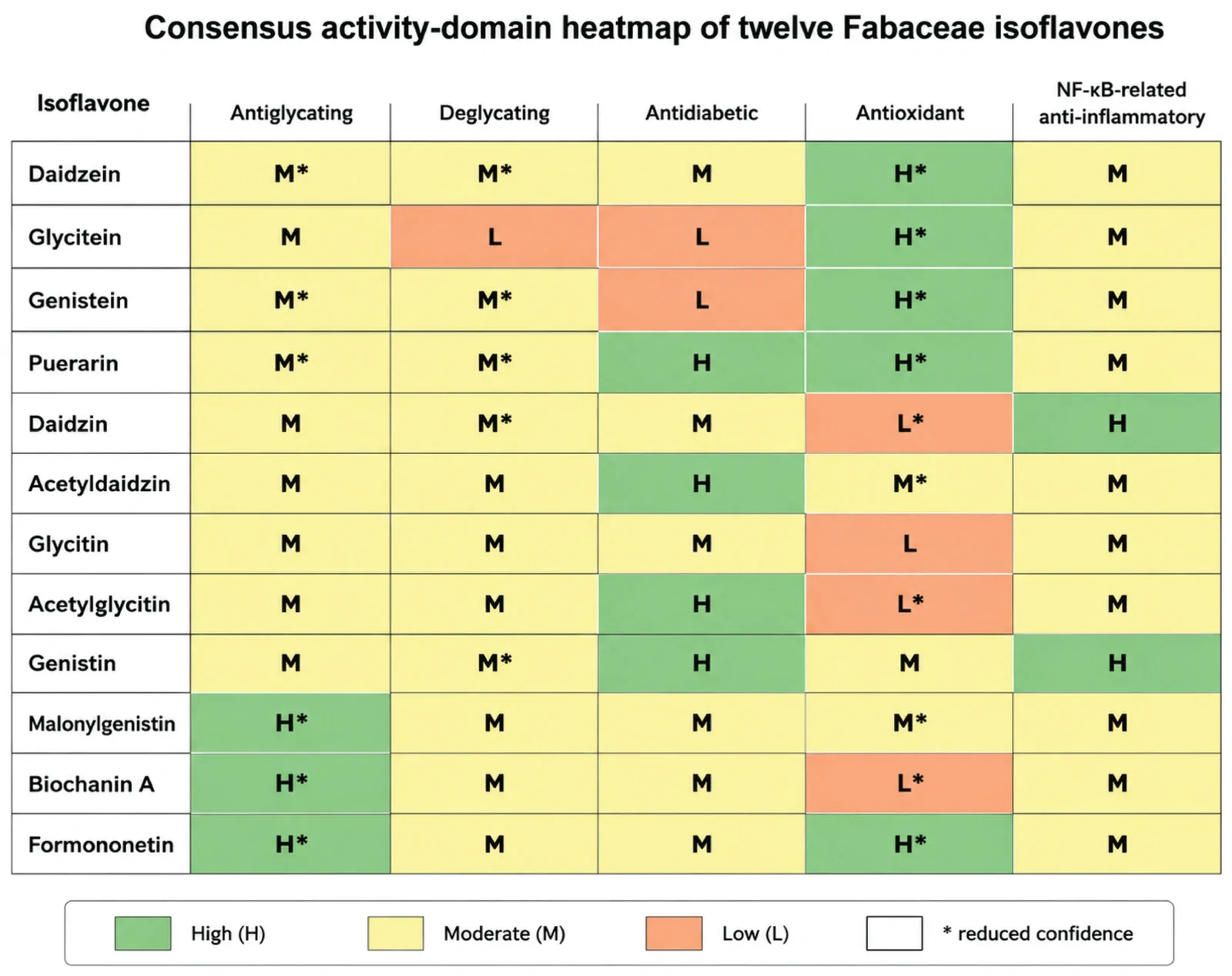
Unified heatmap matrix of the final consensus pharmacodynamic classification of the twelve selected Fabaceae isoflavones across five diabetes-relevant activity domains. Rows represent individual isoflavones, and columns represent antiglycating, deglycating, antidiabetic, antioxidant, and NF-κB-related anti-inflammatory activities. The cell colors indicate the final consensus activity category: high (H), moderate (M), or low (L). An asterisk (*) indicates reduced confidence in the assigned category due to partial divergence or incomplete concordance among the computational outputs used for that domain.

To facilitate direct comparison of the final categorical outputs across all compounds and activity domains, the consensus results are summarized as a unified heatmap matrix in Figure 3.

The heatmap highlights the domain-specific nature of prioritization. No single compound was superior across all five evaluated domains. Instead, malonylgenistin, biochanin A, and formononetin were associated mainly with antiglycating potential; acetylglycitin, puerarin, genistin, and acetyldaidzin, with predicted antidiabetic activity; genistein, with antioxidant potential; and daidzin and genistin, with NF-κB-related anti-inflammatory potential.

### 2.8. Pharmacokinetic and Toxicity Properties

The predicted absorption profiles clearly separated the isoflavone glycosides/conjugates (genistin, acetyldaidzin, malonylgenistin, glycitin, acetylglycitin, and puerarin) from the corresponding aglycones (daidzein, genistein, glycitein, formononetin, and biochanin A) (Table 8). Most glycosylated derivatives displayed Caco-2 permeability coefficients below the high-permeability cutoff (log Papp < −5.1, i.e., Papp < 8 × 10^−6^ cm/s), together with low predicted human oral bioavailability at both the 20% and 50% thresholds, whereas the aglycones consistently exceeded this cutoff and were classified as bioavailable, in agreement with the well-documented requirement for pre-absorptive hydrolysis of isoflavone glycosides by intestinal β-glucosidases prior to systemic uptake. This trend was not fully uniform at the 20% bioavailability threshold, however: daidzin was the sole glycoside classified as bioavailable at this lower threshold (low confidence), while remaining non-bioavailable at the more stringent 50% threshold. This partial discordance indicates that the glycoside/aglycone separation in predicted oral bioavailability is directionally consistent—and fully concordant with the Caco-2 permeability data—but is not absolute across every compound and every confidence tier and should therefore be interpreted at the level of the overall structural trend rather than as a categorical rule applicable to each individual glycoside. Human intestinal absorption was predicted to be favorable (>30%) for most compounds, with malonylgenistin and puerarin being notable exceptions (nonabsorbed, low–medium confidence). None of the compounds were predicted P-glycoprotein substrates with high confidence, and only formononetin and glycitein were flagged as potential P-glycoprotein inhibitors (high confidence); given the generally low-to-medium confidence associated with several of these calls, these absorption estimates should be regarded as indicative rather than conclusive and would benefit from confirmation using an experimental Caco-2 or PAMPA permeability assay.

Predicted blood–brain barrier permeability (log PS) followed the same glycoside/aglycone dichotomy observed for absorption: most glycosylated isoflavones yielded log PS values below the Central Nervous System-negative threshold (≤−3) and were consistently classified as non-penetrant (high confidence), while the aglycones showed higher log PS values approaching or exceeding the Central Nervous System-positive threshold (≥−2), with formononetin and biochanin A predicted as blood–brain barrier penetrant, albeit with low-to-medium confidence. One glycoside, acetyldaidzin, was also classified as penetrant (high confidence) despite a log PS value of −3.75 within the range otherwise associated with non-penetrant glycosides, representing an exception to the glycoside/aglycone dichotomy and, like the genistein discordance noted below, illustrating the non-linear nature of the classification boundary used by the underlying model. Genistein and daidzein, in contrast, were predicted as nonpenetrant despite comparable log PS values, illustrating the model’s nonlinear decision boundary and reinforcing the need for cautious interpretation of borderline cases. Plasma protein binding was predicted to be moderate-to-high for all the compounds (74.6–92.3%), which is consistent with the polyphenolic character of isoflavones. The corresponding fraction unbound values (0.82–1.40) are reported on the Deep-PK model’s native regression scale rather than as the literal complement of plasma protein binding and should not be read as the numerical unbound proportion of drug in plasma; they are used here only for relative, within-panel comparison. The fraction unbound values and steady-state volumes of distribution were both noticeably lower for the aglycones (0.30–0.48 L/kg) than for the glycosylated forms (0.77–0.93 L/kg), suggesting a more restricted predicted tissue distribution for the deglycosylated metabolites, possibly reflecting their greater plasma protein affinity (Table 9).

The in silico metabolism panel indicated that cytochrome P450-mediated interactions were largely confined to the aglycone forms (Table 10). Biochanin A, formononetin, genistein, glycitein, and daidzein were each predicted to inhibit multiple major CYP isoforms, most consistently CYP1A2, CYP2C19, and CYP3A4, with formononetin additionally flagged as a CYP2D6 substrate and a CYP1A2 substrate and biochanin A/glycitein as a CYP2D6 inhibitor; none of the glycosylated conjugates showed comparable CYP inhibitory potential. Conversely, the substrate status of CYP2C9 was predicted almost universally across the panel (11 of 12 compounds), suggesting that this isoform may play a role in the oxidative metabolism of both glycosides and aglycones. Breast cancer Resistance protein inhibition was predicted for several aglycones as well as for malonylgenistin and acetylglycitin, whereas none of the compounds were predicted to inhibit OATP1B1 or OATP1B3. Collectively, these results point to the potential for CYP-mediated drug–isoflavone interactions restricted mainly to the aglycone metabolites, a hypothesis that would require confirmation through in vitro CYP inhibition assays before any pharmacokinetic interaction can be inferred with confidence.

The predicted total clearance was systematically greater for the glycosylated isoflavones (7.30–11.18 mL/min/kg) than for their aglycone counterparts (3.04–4.55 mL/min/kg), suggesting a comparatively faster predicted elimination of the intact conjugates, which may in part reflect their more hydrophilic character (Table 11). All twelve compounds were classified as having a short elimination half-life (<3 h), although the confidence associated with this classification was generally low for the glycosides and medium for the aglycones. Notably, Deep-PK reports elimination half-life as a binary categorical output (<3 h vs. ≥3 h) rather than as a continuous point estimate; consequently, the half-life classification cannot be directly cross-validated against the numerical clearance values in the same table using standard pharmacokinetic relationships (e.g., t½ = 0.693 × Vd/CL), since no continuous half-life value is available from the model, and the two endpoints represent independent predictions rather than internally derived quantities. Given the short predicted half-lives across the entire series, the plasma persistence of these isoflavones in vivo is likely to depend substantially on continuous intestinal absorption/enterohepatic recirculation rather than on slow systemic elimination, a point that in silico clearance and half-life predictions alone cannot resolve and that warrants experimental pharmacokinetic verification.

The toxicity predictions revealed a consistent pattern in which the Ames mutagenicity outcome mirrored the glycoside/aglycone divide observed for the ADME parameters: all seven glycosylated isoflavones were predicted to be mutagenic (high confidence), whereas all five aglycones were predicted to be nonmutagenic (medium-to-high confidence) (Table 12). This finding is at odds with the substantial body of experimental and epidemiological evidence supporting the safety of dietary isoflavone glycosides and should therefore be interpreted with particular caution, likely reflecting a limitation of the underlying training data or structural applicability domain of the model rather than genuine genotoxic potential; experimental Ames testing is recommended before any conclusions are drawn. Carcinogenicity was predicted to be low for nearly the entire panel, with genistein being the only exception (toxicity, low confidence). In contrast, hepatotoxicity predictions (Liver Injury I and II) tended in the opposite direction of the mutagenicity results: the glycosylated conjugates were largely classified as safe for Liver Injury I (low-to-medium confidence), whereas all five aglycones were flagged as hepatotoxic; for Liver Injury II, nearly the entire compound set, glycosides and aglycones alike, was classified as toxic (medium confidence, except genistein, low confidence). Given the generally low-to-medium confidence levels associated with several of these toxicity endpoints and the apparent dietary safety data for isoflavone glycosides, these in silico toxicity flags should be regarded as hypothesis-generating rather than definitive, and any conclusions concerning the safety of these compounds should ultimately rely on dedicated in vitro/in vivo toxicological assessments.

## 3. Discussion

The present study applied a consensus in silico strategy to compare twelve structurally related Fabaceae isoflavones across five pharmacodynamic domains relevant to type 2 diabetes mellitus: antiglycating, deglycating, hypoglycemic/antidiabetic, antioxidant, and NF-κB-related anti-inflammatory activity. Rather than identifying a single compound with superior predicted activity across all domains, the analysis demonstrated that different isoflavones were preferentially associated with different pharmacodynamic mechanisms. These findings indicate that Fabaceae isoflavones should be considered a chemically related but pharmacodynamically heterogeneous group rather than interchangeable compounds.

The absence of completely inactive compounds should also be interpreted in the context of their shared isoflavone/chromone scaffold with phenolic substitution, a structural framework commonly associated with broad low-to-moderate biological reactivity, including antioxidant and signaling-related effects. Consequently, the principal contribution of the present screening lies not in demonstrating that these compounds possess biological activity but in distinguishing their relative pharmacodynamic profiles and providing a rational basis for mechanism-specific experimental prioritization.

This interpretation is consistent with the broader literature describing isoflavones as pleiotropic natural products capable of affecting oxidative stress, inflammation, glucose metabolism, and cellular stress responses [7,9,17,19,20]. However, the present work provides different types of contributions. Many previous studies have focused on individual isoflavones, isolated biological targets, or single pharmacological endpoints [7,9,17,19,20]. In contrast, the current study compared a coherent panel of aglycones, C-glycosides, O-glycosides, acylated glycosides, and methoxylated aglycones using the same integrated computational framework. Therefore, the novelty of this work lies in the comparative, mechanism-oriented prioritization of individual isoflavones across several pathways relevant to diabetes.

Although deglycating activity is less frequently investigated than antiglycating activity in antidiabetic drug discovery, its inclusion is biologically justified because protein glycation is a major contributor to the development of diabetic complications. Chronic hyperglycemia promotes the nonenzymatic glycation of proteins, nucleic acids, and lipids, leading to the accumulation of AGEs, which contribute to oxidative stress, inflammation, endothelial dysfunction, and tissue damage through AGE-dependent signaling pathways [21,22].

Importantly, antiglycation and deglycation represent distinct biological mechanisms. Antiglycation prevents or inhibits the formation of early glycation products, whereas deglycation involves the repair or removal of reversible early glycation adducts before irreversible AGE formation occurs. Once stable AGEs have formed, repair is no longer possible, and damaged proteins must instead be eliminated through normal protein turnover. Therefore, antiglycation and deglycation represent complementary rather than interchangeable protective mechanisms against glycation-induced damage [21,22,23,24].

Accordingly, evaluating both predicted antiglycating and deglycating activities provides a broader assessment of the potential of candidate compounds to interfere with glycation-associated pathology. Although deglycation remains a comparatively underexplored pharmacological endpoint, it represents a biologically meaningful mechanism that complements conventional antiglycation strategies for limiting diabetes-related complications [21,22,23,24].

The observed distinction between the predicted antiglycating and deglycating profiles is biologically meaningful and is consistent with the general concept that phenolic compounds primarily interfere with the early stages of glycation through multiple mechanisms, including the reduction in oxidative stress, the trapping of reactive carbonyl species, and the modulation of protein glycation pathways [25,26,27,28]. Nevertheless, no compound was classified as highly deglycating, suggesting that the selected isoflavones may be more promising for limiting glycation or AGE formation than for reversing already established glycation products. Therefore, antiglycating and deglycating effects should be validated separately using appropriate albumin-, collagen-, hemoglobin-, or AGE-crosslink-based assays.

The high ranking of puerarin is biologically plausible because this compound is widely studied among *Pueraria* isoflavones and has been associated with glucose and lipid metabolism, insulin resistance, and inflammatory regulation in experimental models [17,19]. The prioritization of genistin and acylated glycosides also highlights the possible importance of glycosidic and acylated substitution patterns in the computational recognition of antidiabetic potential. However, this should not be interpreted as direct evidence that these derivatives will be more effective in vivo. Isoflavone aglycones, O-glycosides, C-glycosides, and acylated glycosides may differ considerably in terms of solubility, intestinal absorption, microbial metabolism, conjugation, and systemic exposure [16].

An important caveat applies specifically to the glycosidic derivatives prioritized in the antidiabetic (acetylglycitin, puerarin, genistin, acetyldaidzin) and NF-κB-related (daidzin, genistin) domains. With the partial exception of puerarin, whose C-glycosidic bond is resistant to intestinal β-glucosidase and microbial hydrolysis and which shows demonstrable oral absorption in vivo [21], O-glycosidic isoflavones are extensively hydrolyzed by intestinal mucosal and microbial β-glucosidases prior to systemic absorption such that the aglycone (and its phase-II glucuronide/sulfate conjugates) rather than the intact glycoside is the dominant circulating and tissue-exposed species [17]. This matters for the present predictions, specifically because hydrolysis removes the sugar moiety that contributes much of the structural bulk, polarity, and hydrogen-bonding pattern used by the similarity-based (IT Microcosm, Microcosm BioS) and docking-based models to recognize a compound as active; a favorable predicted pose or descriptor neighborhood for daidzin or genistin therefore does not necessarily transfer to the released aglycone, daidzein or genistein, and vice versa. The effect can be compounded further downstream, since aglycones such as daidzein are themselves substrates for additional gut-microbial biotransformation—most notably conversion to equol and related metabolites—which can yield circulating species with substantially different receptor affinity and redox behavior than the parent compound modeled here and whose formation is highly variable between individuals (“equol-producer” versus “nonproducer” phenotypes) [21,22]. Puerarin’s C-glycosidic bond avoids the hydrolysis step itself, although its low aqueous solubility and limited passive permeability independently constrain oral bioavailability [23]. The present docking- and similarity-based predictions for genistin and daidzin against intracellular NF-κB-related targets should therefore be interpreted as descriptors of intrinsic structure-based target complementarity of the administered form rather than as evidence of achievable in vivo target engagement by the intact glycoside. We recommend that experimental validation of these candidates include the corresponding aglycones and predicted microbial metabolites alongside the parent compounds and that pharmacokinetic profiling be prioritized before any glycoside-specific mechanistic claim is advanced.

The classification of genistein illustrates the value of a multi-endpoint approach. Although genistein is widely discussed in the literature as a biologically active isoflavone with relevance to oxidative stress, inflammation, β-cell protection, and metabolic regulation, it was not prioritized as a high antidiabetic candidate in the present consensus model [9,20,24]. This should not be interpreted as evidence against its antidiabetic relevance. Rather, these findings indicate that, within the specific computational framework used here, genistein was more strongly represented as an antioxidant candidate than as a direct hypoglycemic/antidiabetic candidate. This difference highlights how the same molecule may be favorable in one pharmacodynamic domain but less prominent in another.

The partial difference between similarity-based and quantum-chemical predictions highlights the complexity of antioxidant activity assessment. Because antioxidant activity is strongly method dependent, the predicted rankings may vary according to the assay system, radical species, reaction medium, and biological model used [27,29]. Structural similarity to known antioxidant compounds does not necessarily correspond to thermodynamic favorability in a radical-reaction model, and neither approach fully substitutes for experimental antioxidant assays. For this reason, the uncertainty marks should be retained in the final interpretation. Antioxidant candidates should be evaluated using both chemical assays and cell-based oxidative stress models.

NF-κB-related analysis illustrates the value of integrating similarity-based prediction with molecular docking when candidate compounds are prioritized. These findings are consistent with published evidence that isoflavones can modulate inflammatory signaling pathways, including NF-κB-associated mechanisms. However, the interpretation of the docking-derived outputs requires caution. In the present study, molecular docking suggested favorable binding profiles for all twelve compounds, whereas the similarity-based indices provided greater discrimination among the compounds. This difference supports the use of a consensus approach rather than relying on docking alone. Molecular docking estimates potential ligand–target affinity under simplified assumptions but does not confirm cellular target engagement, transcriptional inhibition, cytokine suppression, or pathway modulation [30]. Therefore, the prioritized compounds identified by the consensus analysis should be considered candidates for subsequent NF-κB-related experimental validation rather than confirmed NF-κB inhibitors.

Notably, the present docking analysis was designed as a consensus affinity-ranking screen across the full compound panel, generating a mean docking energy and derived affinity index for every isoflavone against all nine validated NF-κB models (Table 6), rather than as an exhaustive residue-level interaction study of each compound. Detailed visualization of ligand–residue interactions (Figure 2) was limited to the two compounds that achieved the highest final consensus NF-κB-related scores, namely, genistin and daidzin, for which key stabilizing residues (Gln241, Arg187, and Tyr188) were identified in the representative docked poses. Comprehensive interaction-level mapping for the remaining compounds in the panel was outside the scope of this prioritization-oriented screen and is recommended as part of the proposed follow-up structure-based validation (e.g., extended pose analysis, cocrystallographic studies, or focused docking refinement) for the compounds identified here as domain-specific priority candidates.

Taken together, the results support a domain-specific validation strategy rather than the selection of a single universally superior isoflavone. The observed pharmacodynamic heterogeneity suggests that different Fabaceae isoflavones may contribute to complementary biological mechanisms, including antiglycation, glucose homeostasis, antioxidant defense, and NF-κB-related inflammatory regulation. This distribution may help explain why isoflavone-rich Fabaceae extracts frequently exhibit broad biological effects, with individual constituents contributing to different mechanistic components of the overall activity.

The present study has several limitations. First, all findings are derived from computational models and should therefore be regarded as hypothesis-generating rather than as evidence of biological efficacy. A high predicted score, favorable similarity index, or strong docking-derived affinity does not indicate pharmacological activity, target engagement, or therapeutic effectiveness.

Second, the ligand-based prediction methods used in this study, including PASS, IT Microcosm, and similarity-based approaches, infer biological activity primarily from the chemical structure of the investigated compounds. Such methods are particularly suitable for the early-stage prioritization of natural products because the evaluated pharmacodynamic endpoints (antiglycating, deglycating, antidiabetic, antioxidant, and NF-κB-related anti-inflammatory activities) represent complex systemic and multitarget biological processes rather than the modulation of a single molecular target. However, these approaches also have inherent limitations. They rely predominantly on two-dimensional molecular representations and on the quality and diversity of the reference compounds used for model development. Consequently, they do not explicitly account for three-dimensional protein structures, protein flexibility, induced-fit effects, or the physicochemical environment governing ligand–target interactions, which may influence biological activity.

To partially address these limitations, the present study combined complementary computational approaches, including ligand-based prediction, quantum-chemical modeling, molecular docking specifically for the NF-κB-related anti-inflammatory analysis, and in silico ADMET analysis. The consensus integration of independent computational methods reduces the reliance on any single prediction strategy and provides a more robust basis for comparative compound prioritization. Nevertheless, the consensus framework remains a computational prioritization tool and cannot replace experimental validation.

Third, although in silico ADMET prediction was incorporated to complement the pharmacodynamic assessment, these predictions remain computational estimates and cannot substitute for experimental pharmacokinetic or toxicological evaluation. Parameters such as intestinal absorption, metabolism, tissue distribution, plasma protein binding, and toxicity should therefore be interpreted cautiously and verified experimentally before biological conclusions are drawn. This is particularly important for isoflavones because glycosylation, acylation, intestinal hydrolysis, and microbial biotransformation substantially influence systemic exposure and biological activity. In particular, the compound-level exceptions noted for daidzin (20% oral bioavailability) and acetyldaidzin (BBB penetration) underscore that the glycoside/aglycone dichotomy observed across most ADMET endpoints should be treated as a general trend rather than a categorical rule, and that individual predictions—especially those with low or medium confidence—should be verified independently before being used to support compound-specific pharmacokinetic claims.

Fourth, molecular docking simplifies the biological environment and may not adequately account for protein flexibility, solvent effects, pH, competing ligands, intracellular concentrations, or downstream signaling complexity. Similarly, the quantum-chemical antioxidant classification relied on the semiempirical PM7 method without explicit solvent modeling. Although this approach is appropriate for relative comparison across structurally related compounds, the calculated Gibbs free energy values should not be interpreted as quantitatively exact thermodynamic parameters. Confirmation using higher-level density functional theory methods (e.g., M06-2X or ωB97X-D with an implicit solvation model such as SMD) would further strengthen future antioxidant assessments.

Finally, independent prediction methods were expressed using a categorical consensus framework supplemented by explicit percent agreement values rather than continuous statistical measures of intermethod variability. Future developments may incorporate normalized rank-aggregation approaches (e.g., Borda count or rank-by-rank consensus) together with experimental benchmark datasets to further improve the robustness and validation of consensus-based prioritization.

Future work should proceed through endpoint-specific validation of the compounds prioritized by the consensus analysis. Antiglycation candidates should be evaluated using established protein glycation models, whereas compounds prioritized for antidiabetic activity should be investigated in glucose metabolism, enzyme inhibition, insulin resistance, and β-cell protection assays. The predicted antioxidant candidates should be validated using complementary chemical assays, higher-level DFT calculations with implicit solvation, and cell-based oxidative stress models. Likewise, NF-κB-prioritized compounds should be examined using reporter assays, cytokine expression analysis, and inflammatory marker evaluation. In parallel, the in silico ADMET predictions presented in this study should be complemented by experimental pharmacokinetic and toxicological investigations to verify the predicted absorption, metabolism, and safety profiles before advancing the most promising candidates to in vivo studies.

To conclude, this study provides a structured computational basis for selecting individual Fabaceae isoflavones for further diabetes-related biological validation. Its main value lies in comparative prioritization rather than experimental confirmation. These findings support the view that structurally related isoflavones may contribute to antiglycating, antidiabetic, antioxidant, and anti-inflammatory mechanisms and that consensus in silico screening can serve as a useful first step in mechanism-oriented natural product research.

## 4. Materials and Methods

### 4.1. Selection of Isoflavones

A panel of twelve representative isoflavones was selected to provide a chemically coherent yet structurally diverse dataset for consensus in silico pharmacodynamic screening. Compound selection was based on three main criteria: relevance to Fabaceae-derived phytochemicals, occurrence in isoflavone-rich botanical sources, and suitability for comparative evaluation across pharmacodynamic mechanisms associated with T2DM. The selected panel was designed to move beyond the assessment of isolated aglycones and to include glycosylated, acylated, and methoxylated derivatives, thereby allowing the influence of substitution pattern and structural class on predicted activity to be considered.

The compounds investigated were daidzein, glycitein, genistein, puerarin, daidzin, acetyldaidzin, glycitin, acetylglycitin, genistin, malonylgenistin, biochanin A, and formononetin. These molecules represent major structural subclasses of naturally occurring Fabaceae isoflavones, including aglycones, C-glycosides, O-glycosides, acetylated O-glycosides, malonylated O-glycosides, and methoxylated aglycones. These compounds have been reported in Fabaceae-derived materials, particularly soybean, *Pueraria* spp., and *Trifolium pratense* [31,32,33,34,35,36,37,38,39,40]. Detailed structural information, Fabaceae occurrence, and rationale for inclusion of the twelve selected Fabaceae isoflavones used for in silico screening are provided in Table S2.

The inclusion of daidzein, glycitein, and genistein enabled comparison of parent aglycone structures, whereas puerarin was included as a representative C-glycoside. Daidzin, glycitin, and genistin were selected as O-glycosidic derivatives, while acetyldaidzin, acetylglycitin, and malonylgenistin were included to address the possible contribution of acylation to the predicted pharmacodynamic behavior. Biochanin A and formononetin were incorporated as methoxylated aglycones to compare methylated structures with their corresponding nonmethylated analogs.

**Figure 4:**
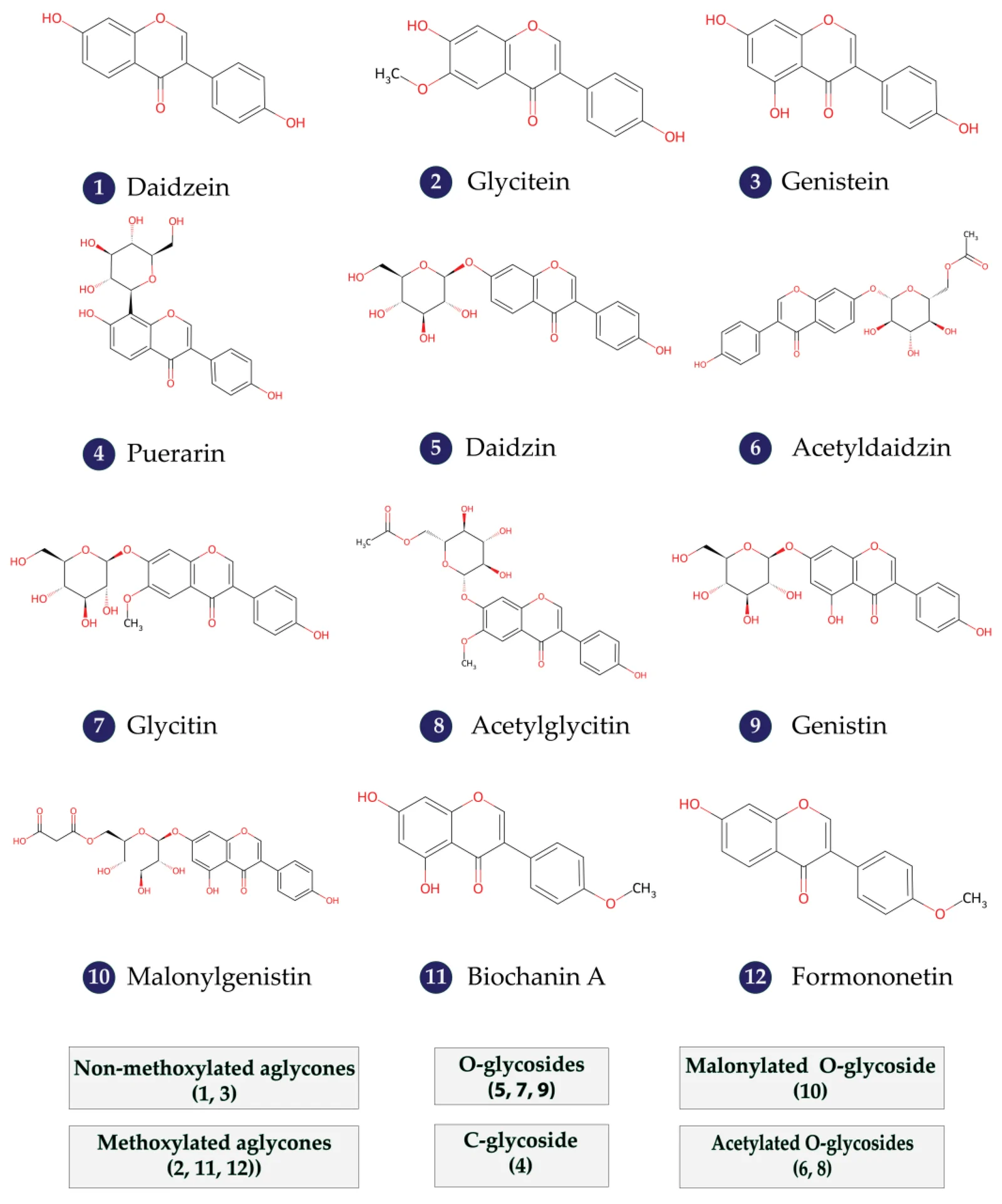
Chemical structures and structural classification of the twelve isoflavone compounds included in the in silico screening study. The selected panel includes aglycone isoflavones, C-glycosides, O-glycosides, acetylated O-glycosides, malonylated O-glycosides, and methoxylated aglycones representative of Fabaceae isoflavones.

On the basis of these considerations, the final compound set provided a balanced structural basis for evaluating how aglycone/glycoside status, acylation, C- versus O-glycosidic linkage, and methoxylation may influence predicted antiglycating, deglycating, antidiabetic, antioxidant, and NF-κB-related activity. The chemical structures and structural classification of the twelve selected compounds are presented in Figure 4.

### 4.2. Molecular Structure Preparation

The two-dimensional chemical structures of the twelve selected isoflavones were prepared as the initial input dataset for subsequent computational analysis. Each structure was checked for chemical correctness, including atom connectivity, bond order, valence state, stereochemical consistency where applicable, and agreement between the compound name and molecular structure. This step was essential to ensure that all subsequent predictions were based on standardized and comparable chemical representations.

For descriptor-based prediction, the molecular structures were converted into standard chemical file formats compatible with the applied in silico platforms. Structures were prepared using ChemOffice/ChemFinder 20.1.1 and Chemaxon Marvin/MarvinSketch 17.1.23 [41], and the resulting files were saved in structured data files (SDF) or MDL information system (MOL) format for subsequent computational workflows.

For calculations requiring three-dimensional molecular geometry, the two-dimensional structures were converted into 3D conformations. Hydrogen atoms were added, and the resulting structures were inspected to confirm a chemically reasonable geometry before optimization. Several conformers were generated for each compound, using Marvin/MarvinSketch, and the lowest-energy conformer was selected for subsequent calculations. For quantum-chemical antioxidant modeling, both the parent isoflavones and their proposed hydroxyl-radical oxidation products were prepared using the same procedure. For molecular docking, the three-dimensional ligand structures were energy-minimized in PyRx 0.8 using the integrated Open Babel module. The optimized three-dimensional ligand structures with added hydrogen atoms were imported into PyRx 0.8, assigned AutoDock-compatible atom types and Gasteiger partial charges, and converted to PDBQT format.

The standardized molecular structures were subsequently used as input data for IT Microcosm-based prediction, PASS analysis, similarity-based antioxidant prediction, quantum-chemical modeling, and NF-κB-related docking. A summary of the computational platforms, input data, outputs, and their roles in the consensus workflow is provided in Table S3.

### 4.3. Prediction of Antiglycating and Deglycating Activities Using an IT Microcosm

The predicted antiglycating and deglycating activities of the selected isoflavones were evaluated using the IT Microcosm v.7.2 computational platform [42]. This method was adopted because it enables structure-based prediction of pharmacological activity through descriptor-based comparisons with reference datasets of compounds with previously established experimental activity.

The standardized molecular structures of the twelve isoflavones were imported into the IT Microcosm workflow and translated into QL structural descriptors. The QL descriptor system provides a hierarchical representation of the chemical structure, including atom-centered fragments, bond characteristics, path–length relationships, and higher-order combinations of structural fragments. These descriptors were used to compare the investigated isoflavones with reference datasets containing compounds classified according to antiglycating and deglycating activity. The reference antiglycating and deglycating datasets were derived from curated bioactivity data sources, including ChEMBL [43,44].

Predictions were performed separately for antiglycating and deglycating activity. For each endpoint, the software generated categorical outputs corresponding to different activity levels. The activity levels were expressed as high, moderate, low, or inactive, depending on the agreement between the prediction strategies. Internally inconsistent or contradictory outputs were treated as prediction refusal and were marked accordingly.

Three complementary prediction strategies were applied: conservative, normal, and risk-based. A conservative strategy was used to identify the most stable and reproducible structure–activity patterns. The normal strategy allowed consideration of both common and less frequent structural features associated with activity. The risk-based strategy was included to account for potentially relevant but less conventional structural patterns, particularly in cases where novel or less represented molecular features may contribute to activity.

The IT Microcosm prediction framework has been described previously; therefore, only the decision rules used for activity classification are summarized here:

High = Ahigh + Ahigh/moderate + Aactive

Moderate = Nhigh + Ahigh/moderate + Aactive

Low = Nhigh + Nhigh/moderate + Aactive

Inactive = Nhigh + Nhigh/moderate + Nactive

where A indicates a positive prediction and N indicates a negative prediction for the corresponding activity level.

For each activity endpoint, the outputs obtained from the three strategies were integrated into a final consensus classification. A compound was assigned to the High category when at least one strategy indicated high predicted activity and the remaining outputs did not contradict this assignment. A moderate category was assigned when the dominant reliable pattern indicated moderate predicted activity. A low category was assigned when the available outputs pointed to weakly predicted activity. When the prediction pattern was incomplete or partially divergent, a question mark was added to indicate reduced confidence in the final classification.

The final output of this stage consisted of compound-specific consensus predictions for antiglycating and deglycating activities. These outputs were subsequently integrated into the broader multifunctional pharmacodynamic profile of the twelve isoflavones.

### 4.4. Prediction of Hypoglycemic and Antidiabetic Activity

The predicted hypoglycemic and antidiabetic activities of the twelve selected isoflavones were evaluated using PASS 10.4 Professional Extended [45,46]. PASS was applied to estimate the probability that each compound may possess biological activities relevant to glucose regulation and diabetes-associated pharmacodynamic effects on the basis of its chemical structure. Molecular docking was not used for antidiabetic activity prediction; docking was reserved exclusively for the NF-κB-related anti-inflammatory analysis described below.

The prepared molecular structures were subjected to PASS analysis, and two activity categories were selected: hypoglycemic activity and antidiabetic activity. For each compound and each activity category, the PASS method generated two probability values: Pa, which represents the probability of activity, and Pi, which represents the probability of inactivity. A compound was considered to have predicted activity when Pa exceeded Pi.

To enable quantitative comparisons between compounds, the Pa/Pi ratio was calculated for each activity. Higher Pa/Pi values were interpreted as indicating a stronger predicted likelihood of the corresponding activity. The Pa/Pi values were then converted into an activity index according to predefined activity ranges:

R_A,C_ = Pa_A,C_/Pi_A,C_ (1)

where R_A,C_ is the likelihood ratio for activity A in compound C, Pa_A,C_ is the probability of activity, and Pi_A,C_ is the probability of inactivity.

The hypoglycemic and antidiabetic indices were subsequently combined to obtain an overall PASS-derived estimate of diabetes-related activity for each compound.

Ind_PASS_ = (Ind_hypoglycemic_ + Ind_antidiabetic_)/2 (2)

where Ind_PASS_ represents the integrated PASS-derived index of diabetes-related activity.

The PASS-derived outputs included the Pa/Pi values, activity indices, and predicted activity categories for each isoflavone. Compounds with higher combined indices were considered to have stronger predicted hypoglycemic/antidiabetic potential. To obtain the final consensus antidiabetic profile, the PASS-derived index was subsequently integrated with the IT Microcosm-derived index.

IndC = (IndM + IndP)/2(3)

where Indc is the final consensus antidiabetic index, IndM is the IT Microcosm-derived index, and Ind P is the PASS-derived index.

### 4.5. Similarity-Based Prediction of Antioxidant Activity

The antioxidant activity of the selected isoflavones was first evaluated using a “similarity-based prediction approach” implemented in the TestSim 7.2 module of IT Microcosm. This method was used to estimate the antioxidant potential by comparing the QL descriptor profile of each isoflavone with reference compounds previously characterized for antioxidant activity.

The molecular structures were translated into QL structural descriptors and compared with a reference database of experimentally studied antioxidant compounds. Structural similarity between each investigated isoflavone and the reference compounds was assessed using the QL-modified Tanimoto similarity coefficient [47]. The general form of the Tanimoto coefficient is

T = c/(a + b − c)(4)

where T is the Tanimoto similarity coefficient, a is the number of descriptors present in compound 1, b is the number of descriptors present in compound 2, and c is the number of descriptors shared by both compounds.

In the IT Microcosm/TestSim workflow, this calculation was adapted to QL structural descriptors. For each isoflavone, the maximum similarity value to antioxidant reference compounds was recorded as the TQL. This value was used as a measure of structural resemblance to compounds with known antioxidant activity.

On the basis of the similarity profile and the activity level of the most structurally related reference compounds, each isoflavone was assigned a predicted antioxidant category. The similarity-based output classified compounds as high, moderate, low, or prooxidant. This classification represented the first component of the antioxidant assessment.

Because structural similarity alone does not fully account for radical-scavenging thermodynamics or redox behavior, the similarity-based prediction was subsequently complemented by quantum-chemical modeling of hydroxyl radical oxidation pathways. The two outputs were later integrated to obtain the final consensus antioxidant activity profile.

### 4.6. Quantum-Chemical Modeling of Antioxidant Potential

Quantum-chemical modeling was performed as a complementary approach to the similarity-based antioxidant prediction. This analysis was used to estimate the thermodynamic favorability of the reaction between the selected isoflavones and the hydroxyl radical (•OH), which was selected as a model radical species relevant to antioxidant activity.

For each of the twelve isoflavones, possible oxidation products were generated according to the most likely hydroxyl radical oxidation pathways reported for flavonoid and isoflavonoid structures [48]. On the basis of the chemical structure of the compound, one or two possible oxidation pathways were considered. The parent isoflavones and their corresponding oxidation products were prepared as separate molecular structures for thermodynamic calculations. The compounds assigned to each modeled hydroxyl radical oxidation pathway are summarized in Table 13. The detailed structures of the corresponding oxidation products are provided in Figure S1.

For compounds with two possible oxidation pathways, both reactions were modeled, and the pathway with the lowest Gibbs free energy value was selected for the final antioxidant classification.

Conformational analysis was performed on the parent compounds and oxidation products. For each structure, several conformers were generated, and the conformers with the lowest energy were selected for further optimization. The selected conformers were then optimized using the semiempirical PM7 method in MOPAC. Thermodynamic parameters, including the enthalpy, entropy, and Gibbs free energy, were calculated under standard conditions.

The Gibbs free energy change of the hydroxyl radical oxidation reaction was calculated as the difference between the total Gibbs free energy of the reaction products and the total Gibbs free energy of the initial reactants:

ΔG_rxn_ = ∑ΔG_products_ − ∑ΔG_reactants_ (5)

where ΔGrxn is the Gibbs free energy change of the modeled oxidation reaction.

For compounds with two modeled oxidation pathways, the minimum Gibbs free energy value was selected:

ΔG_min_ = min(ΔG_1_, ΔG_2_) (6)

where ΔG1 and ΔG2 are the Gibbs free energy values of the first and second modeled oxidation pathways, respectively.

The calculated Gibbs free energy values were used to assign relative antioxidant classifications. Cluster-based classification was applied to divide the compounds into three activity categories: above moderate, moderate, and below moderate. The classification rules were as follows:

ΔG_C_ < ΔG_above moderate_ → Above moderate

ΔG_above moderate_ ≤ ΔGC < ΔG_moderate_ → Moderate

ΔG_C_ ≥ ΔG_moderate_ → Below moderate

where ΔGC is the selected Gibbs free energy value for compound C.

It should be noted that semiempirical PM7 calculations, while computationally efficient for panel-wide comparative screening, carry known limitations for the quantitative thermodynamics of open-shell radical reactions relative to higher-level DFT methods. Because all the compounds and their corresponding oxidation products were processed under an identical PM7 protocol, the ΔGmin values were used strictly for relative, tier-based classification (above moderate/moderate/below moderate) rather than as absolute reaction thermodynamics; this is consistent with the ordinal role of this block within the overall consensus scoring framework. The quantum-chemical antioxidant classification was subsequently integrated with similarity-based antioxidant prediction to generate the final consensus antioxidant profile. The modeled oxidation products are shown in Figure S1, and the ΔGMin values used for antioxidant consensus classification are summarized in Table S1.

### 4.7. Prediction of NF-κB-Related Anti-Inflammatory Potential

The predicted anti-inflammatory potential of the selected isoflavones was evaluated in relation to nuclear factor kappa B (NF-κB)-associated signaling. This pathway was selected because NF-κB plays a key role in inflammatory responses associated with insulin resistance, endothelial dysfunction, and diabetic complications [49]. A two-stage computational strategy was adopted, consisting of similarity-based prediction using Microcosm BioS and molecular docking against validated NF-κB-related protein models.

#### 4.7.1. Similarity-Based Prediction Using Microcosm BioS

The first stage of the assessment of NF-κB-related activity was performed using the Microcosm BioS v20.6.6 similarity-based prediction system [42,50]. The prepared molecular structures of the twelve isoflavones were compared with those of reference compounds previously associated with NF-κB inhibition or modulation.

Structural similarity was calculated using the QL-modified Tanimoto similarity coefficient. For each compound, the mean similarity value was recorded as TMean. Importantly, IndMC_BioS is not the raw Tanimoto similarity coefficient itself, but a decile-calibrated ordinal activity index derived from the empirical activity distribution of the reference compound library. The reference database underlying Microcosm BioS contains structure–activity data for 625,888 characterized compounds across 11,509 distinct biological activities [15]; for the NF-κB-related endpoint specifically, R = 2140 experimentally characterized reference compounds were available. For each reference activity, the experimentally determined activity values of all reference compounds were ranked in descending order and partitioned into ten deciles, each assigned an integer index ranging from −5 (lowest decile; inactive) to +5 (highest decile; very highly active), with the index 0 corresponding to the median of the reference distribution. For each investigated isoflavone, the L nearest reference compounds (by QL-modified Tanimoto similarity; L = 10 by default) were identified, and the decile index associated with each nearest reference compound’s experimentally known activity value was retrieved. The compound-specific IndMC_BioS values reported in Table 6 were calculated as the mean decile index across the nearest reference neighbors. This procedure is conceptually a nonparametric, distribution-anchored rank transformation of the raw similarity-based neighbor search, rather than an unscaled or unbounded similarity score, and constrains IndMC_BioS to the same −5-to-+5 range used for the docking-derived index (IndDock; see Section 4.7.2) by construction rather than by post hoc rescaling. On the basis of the activity profile of the nearest reference compounds, an activity index was calculated and expressed as IndMC_BioS. This index was used to estimate the potential ability of each isoflavone to interact with or influence NF-κB-related biological mechanisms.

The same QL-modified Tanimoto similarity principle described in the antioxidant similarity-based prediction section was applied here to identify the nearest reference neighbors. However, in this case, the reference dataset consisted of compounds associated with NF-κB-related activity rather than antioxidant activity, and unlike the purely similarity-ranked antioxidant classification in Section 2.5, the NF-κB-related index additionally incorporates the decile-calibration step described above so that it is directly commensurate with the docking-derived index prior to averaging in Equation (8).

#### 4.7.2. Molecular Docking Protocol

The second stage involved molecular docking to estimate the predicted binding affinity of the selected isoflavones toward NF-κB-related protein models. Docking was performed using prepared three-dimensional ligand structures and NF-κB protein models (listed in Table S4), using AutoDock Vina 1.1.2 as the docking/scoring engine, run within the PyRx 0.8 virtual screening environment. The use of NF-κB structural models, including PDB structures 1NFI and 3GUT, for molecular docking has also been reported in recent studies [51,52,53]. AutoDock Vina calculations were performed using its empirical scoring function, with an exhaustiveness value of 8 and a maximum of 9 output poses per run; no manual reweighting of scoring terms was applied. The docking procedure aimed to evaluate whether the selected isoflavones could form energetically favorable interactions with NF-κB-associated binding sites.

To account for structural variability, an ensemble docking strategy was applied using three structural models for each NF-κB protein/subunit considered in the analysis. The final ensemble comprised three experimental PDB structures used to represent NFKB1 (p50; PDB IDs 2O61, 1SVC, and 3GUT), three ModBase homology models used to represent NFKB2 (p52), and one experimental PDB structure (1NFI) together with two ModBase homology models used to represent RELA (p65). The model identifiers, selected chains where applicable, and docking-grid parameters for all nine models are provided in Table S4.

**Figure 5:**
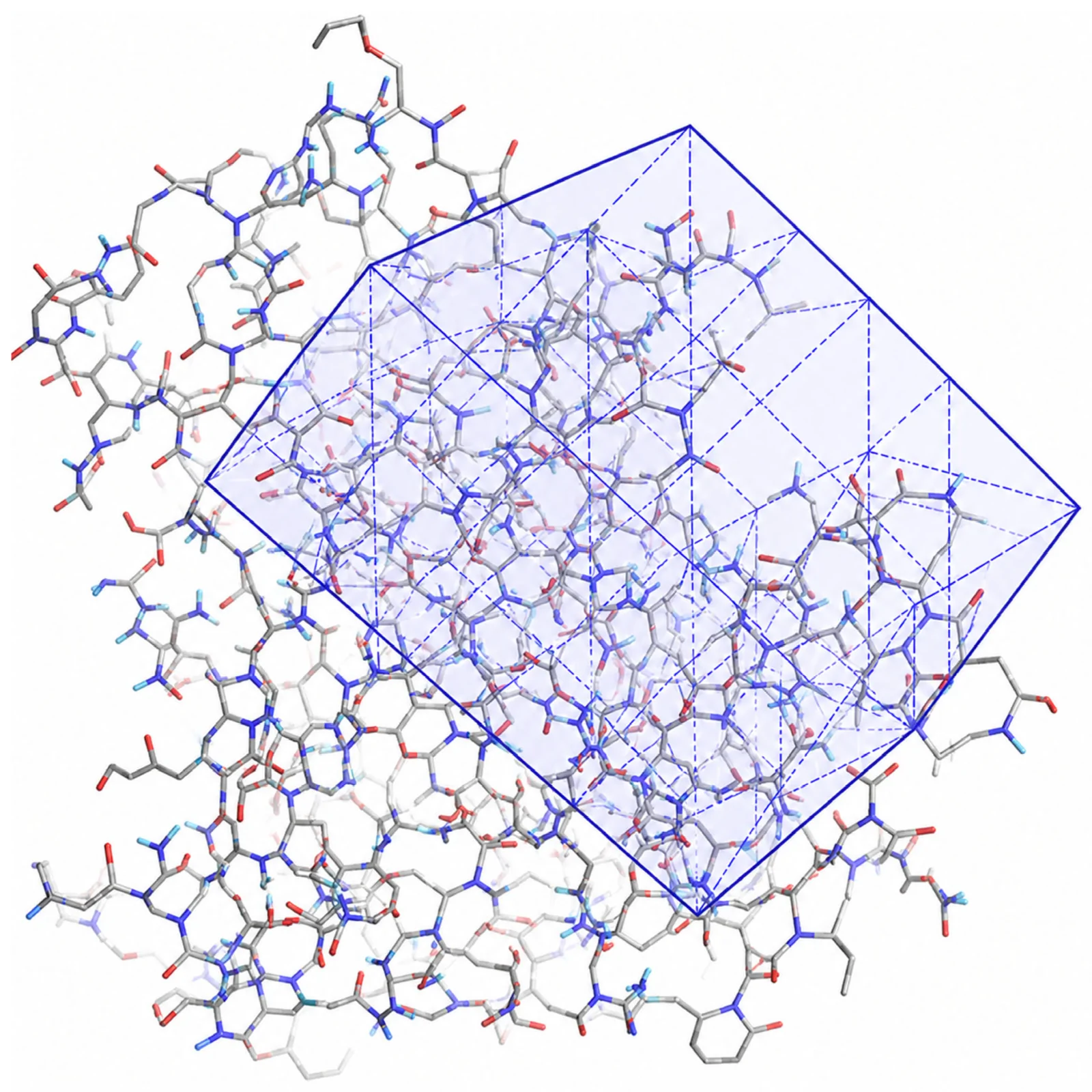
Representative docking grid used for molecular docking of the selected isoflavones into the NF-κB-related binding site in PyRx 0.8 (AutoDock Vina 1.1.2 scoring engine). The wireframe box indicates the docking search space. The grid box was defined independently for each of the nine validated 3D protein models (Section 4.7.2) to fully enclose the previously validated inhibitory binding region.

The docking regions for the nine structural models were defined during the original modeling workflow with reference to available structural and literature information on NF-κB ligand-binding regions. For the representative model illustrated in Figure 5, the key residues contributing to ligand stabilization include Gln241, Arg187, and Tyr188 [51,52,53]. Detailed residue-level interaction analysis was performed only for the two top-ranked compounds selected for representative visualization (Figure 2); for the remaining compounds, the docking output used for consensus scoring was limited to the binding energy (ΔE) and derived affinity index (IndDock).

Before docking, the optimized three-dimensional ligand structures were prepared as described in Section 4.2 and converted to AutoDock-compatible PDBQT format. Rotatable ligand bonds were defined during PDBQT preparation, allowing torsional ligand flexibility during docking. Protein models were constructed by removing nonessential molecules, adding hydrogen atoms, assigning atom types, converting the receptor structures to PDBQT format, and defining the binding regions used for docking. The receptor models were treated as rigid, whereas ligand flexibility was sampled through rotation around the assigned rotatable bonds. Docking was performed using AutoDock Vina 1.1.2 with the default scoring function, an exhaustiveness value of 8, and a maximum of 9 output poses per run. Docking calculations were performed for each ligand, and the best binding poses were selected according to the lowest predicted binding energy [54]. As a single static protein conformation may not fully represent the conformational flexibility of the NF-κB binding site, an ensemble docking strategy was adopted. Docking was performed against nine independently validated 3D structural models spanning three NF-κB isoforms: NF-κB1 (PDB codes 2O61, 1SVC, and 3GUT), NF-κB2 (three homology models), and RELA (p65) (PDB code 1NFI and two homology models). For each ligand–model pair, the docking was repeated five times independently (45 total runs per ligand across the nine models), and the minimum (most favorable) binding energy obtained across replicate runs for each NF-κB isoform was retained; the isoform-level values were then averaged to obtain the mean docking energy (ΔE) reported for each compound. A representative docking grid used for molecular docking into the validated NF-κB binding site is presented in Figure 5.

The docking output was expressed as the mean docking energy (ΔE, kcal/mol). More negative ΔE values were considered to indicate stronger predicted binding affinity. To allow integration with the similarity-based prediction results, the docking energies were converted into a docking-derived activity index IndDock using the following equation:

IndDock = (−10ΔE − 65)/7(7)

where ΔE is the docking energy in kcal/mol.

This transformation was based on the validated docking-energy interval in which ΔE = −10 kcal/mol corresponded to the highest reference affinity and ΔE = −3 kcal/mol corresponded to the lowest reference affinity. Accordingly, the transformed index allowed the docking-derived score to be compared with the Microcosm BioS activity index.

This design choice—anchoring IndDock to the validated reference docking-energy interval (ΔE = −10 to −3 kcal/mol) corresponding to the same experimentally defined active/inactive reference compounds used to calibrate IndMC BioS—ensures that the two indices averaged in Equation (8) are commensurate by construction, both being bounded, reference-population-anchored ordinal scores on an identical −5-to-+5 scale, rather than raw metrics from unrelated mathematical spaces.

For each protein model, the docking grid was centered on the validated NF-κB-related inhibitory/regulatory binding region identified during the model validation procedure. The grid box was adjusted separately for each model to fully include the corresponding binding site residues and to allow ligand conformational sampling within the selected pocket. The complete grid-box parameters, including the center coordinates, grid dimensions, exhaustiveness, and number of generated poses, are provided in Table S4. For the representative RELA (p65) model shown in Figure 5, the grid box was centered at X = 12.458, Y = −3.221, and Z = 27.904, with dimensions of 24.0 × 24.0 × 24.0 Å.

#### 4.7.3. Consensus Classification of NF-κB-Related Activity

The final NF-κB-related activity score was obtained by integrating the similarity-based index (IndMC_BioS) and the docking-derived index (IndDock). The average value was calculated as the integrated consensus index (IndNF-κB) as follows:

Ind_NF−κB_ = (Ind_MC_BioS_ + Ind_Dock_)/2 (8)

On the basis of this integrated index, compounds were assigned to categorical activity levels using predefined thresholds:

Ind_NF−κB_ ≥ 1.5 → High

0 ≤ Ind_NF−κB_ < 1.5 → Moderate

Ind_NF−κB_ < 0 → Low

The final outputs of this stage included the Microcosm BioS-derived similarity index, docking-derived index, integrated NF-κB consensus index, and categorical prediction for each isoflavone. These results were subsequently incorporated into the overall multifunctional pharmacodynamic profile.

### 4.8. Consensus Scoring and Prioritization

A consensus scoring approach was applied to integrate the outputs obtained from the different computational prediction blocks and to generate a comparative multifunctional pharmacodynamic profile for the twelve selected isoflavones. A consensus analysis was performed separately for each biological activity domain, and the results were subsequently combined into an overall activity profile. The evaluated pharmacodynamic domains included antiglycating activity, deglycating activity, hypoglycemic/antidiabetic activity, antioxidant activity, and NF-κB-related anti-inflammatory potential.

The consensus framework intentionally assigned equal weight to each independent computational approach. This decision was based on the fact that the integrated methods represent fundamentally different prediction paradigms, including similarity-based, probability-based, quantum-chemical, and (for the NF-κB-related domain only) molecular docking approaches, each employing different molecular representations, mathematical formalisms, and activity metrics. Because no experimentally validated reference dataset was available to justify objective optimization of method-specific weights, assigning unequal weights would introduce additional subjective bias. Therefore, all the prediction methods were considered independent sources of evidence contributing equally to the consensus classification, which is consistent with the general ensemble-learning (bagging) principle that combining diverse independent predictors improves the robustness of comparative prioritization while reducing the reliance on any single computational method.

For each pharmacodynamic domain, the outputs from all relevant independent computational methods were first converted into common categorical activity levels before consensus integration. Clear agreement between methods supported direct classification, whereas partial disagreement resulted in an uncertainty mark. This approach was used to retain information about prediction confidence and to avoid overinterpretation of inconsistent computational outputs.

The general interpretation rules were as follows:

Agreement between methods → Direct category

Partial disagreement between methods → Category?

Contradictory outputs → Cautious classification or prediction refusal

When computational outputs were partially divergent but still supported assignment to a given activity level, an additional “?” mark was added to the activity category. This mark does not indicate a separate activity level and does not change the assigned category itself. Instead, it indicates reduced confidence in the presence of the assigned activity level because of incomplete concordance among the computational outputs. Thus, “High?” was interpreted as a high predicted activity level with reduced confidence, “Moderate?” as a moderate predicted activity level with reduced confidence, and “Low?” as a low predicted activity level with reduced confidence.

Because the consensus ranking integrates predictions generated using different computational methods, complete agreement among all the methods is not expected. Computational uncertainty was therefore assessed qualitatively through the level of concordance between independent prediction approaches rather than by continuous measures of intermethod variability. The percentage agreement reported for each compound reflects the consistency of the computational evidence supporting its prioritization. Greater agreement indicates greater confidence in the consensus ranking, whereas lower agreement reflects lower confidence rather than evidence against biological activity. Consequently, the consensus ranking should be interpreted as a comparative prioritization framework for selecting compounds for experimental validation rather than as a quantitative measure of pharmacological efficacy.

Agreement was defined as the proportion of independent computational methods supporting the final assigned activity category within each pharmacodynamic domain. To quantify the degree of concordance underlying each categorical assignment, we additionally report a percent agreement (%A) metric, calculated as the proportion of independent prediction strategies/methods within a given domain that support the assigned category out of the total number contributing to that domain’s consensus (e.g., 2/3 IT Microcosm strategies or 1/2 antioxidant prediction approaches). A ‘?’ notation is retained as shorthand for %A < 100% (i.e., partial rather than unanimous concordance) and are always reported together with the corresponding %A values in Table 1, Table 2 and Table 5.

For antiglycating and deglycating activities, the final consensus category was derived from the conservative, normal, and risk-based strategies generated by IT Microcosm. A compound was classified according to the dominant reliable prediction pattern across the three strategies. Contradictory or incomplete outputs were reflected by uncertainty marks where appropriate.

For hypoglycemic/antidiabetic activity, PASS-derived indices and IT Microcosm-derived similarity indices were integrated into a combined consensus index. Compounds with higher integrated indices were assigned to stronger predicted activity categories. The final classification was expressed as high, moderate, or low according to the predefined index thresholds.

For antioxidant activity, two independent outputs were integrated: similarity-based antioxidant prediction and quantum-chemical classification based on hydroxyl radical oxidation modeling. When both methods supported a comparable activity level, the corresponding category was assigned directly. When the outputs diverged, the final category was assigned conservatively and marked with a question mark to indicate reduced confidence.

To determine the anti-inflammatory potential related to NF-κB, the Microcosm BioS similarity-based index and the docking-derived index were integrated into a combined consensus index (IndNF-κB). The final categorical classification was assigned using predefined thresholds: high for IndNF-κB ≥ 1.5, moderate for 0 ≤ IndNF-κB < 1.5, and low for IndNF-κB < 0.

After activity-specific classifications were assigned, all five domains were combined into an integrated multifunctional profile for each compound. Compounds were prioritized in two ways: first, according to their strongest predicted activity in individual domains, and second, according to the breadth of their predicted activity across multiple domains.

Compounds classified as high in at least one pharmacodynamic domain were considered domain-specific priority candidates. Compounds showing at least moderate predicted activity across most domains, with one or more high or potentially high classifications, were considered to have broader multifunctional potential.

The validity of the consensus strategy was evaluated qualitatively by assessing (i) the concordance among independent computational approaches and (ii) the consistency between the consensus-prioritized compounds and published experimental evidence discussed in Section 3. Consensus scoring was used exclusively for comparative prioritization and hypothesis generation and should not be interpreted as an experimental confirmation of biological activity, therapeutic efficacy, or clinical relevance.

### 4.9. Reproducibility and Quality Control

Several quality control measures were applied to improve the transparency and reproducibility of the in silico workflow. Before computational analysis, all molecular structures were checked for consistency of compound names, atom connectivity, bond order, valence states, and structural class. The same standardized structures were used throughout the different computational blocks to ensure comparability between descriptor-based prediction, PASS analysis, antioxidant modeling, and docking-based evaluations.

For descriptor-based prediction, all the structures were converted into compatible molecular file formats before being imported into the corresponding software platforms. The prediction outputs were recorded systematically for each compound and each activity domain. When multiple prediction strategies were used, the individual outputs were retained before consensus classification. This allowed the final activity category to be traced back to the original computational results.

For the PASS-based prediction, both the Pa and the Pi values were recorded for each selected activity. The Pa/Pi ratios, activity indices, and final classifications were calculated using the same rules for all the compounds. This ensured uniform comparison of hypoglycemic and antidiabetic potential across the twelve-isoflavone dataset.

For quantum-chemical modeling, the parent compounds and oxidation products were prepared using the same conformational and optimization workflow. The most energetically favorable conformers were selected for thermodynamic calculations. Gibbs free energy values were recorded for each oxidation pathway, and the lowest-energy pathway was used for classification when more than one oxidation route was available.

The use of semiempirical PM7-derived quantum-chemical parameters as inputs to classification-level structure–activity models has been independently validated within our group’s broader computational pipeline. In parallel screening campaigns applying the same PM7/MOPAC descriptor-generation protocol (parent and reaction-product optimization, conformational sampling, and thermodynamic parameter calculation) to structurally diverse reference compound sets, neural-network QSAR classifiers built on these descriptors—including total molecular energy, heat of formation, and frontier orbital energies (E, H, E_HOMO, E_LUMO, ΔE_HOMO–LUMO)—were assessed by leave-one-out cross-validation (LOOCV) against a training set of 212 compounds with experimentally established activity. This validation yielded high classification performance across accuracy (F_o), sensitivity (F_a), and specificity (F_n) metrics (e.g., F_o = 94.34%, F_a = 100.00%, and F_n = 92.59% for a representative high-activity endpoint). These results indicate that, although semiempirical PM7 calculations carry recognized limitations for the quantitative prediction of absolute open-shell radical reaction thermodynamics relative to higher-level density functional theory (DFT) methods, PM7-derived energetic descriptors retain strong discriminatory power when used for relative, ordinal classification of structurally related compounds—the role they occupy within the present consensus antioxidant assessment (Section 4.6). Accordingly, the ΔGmin values obtained here were treated as a basis for tier-based ranking (above moderate/moderate/below moderate) rather than as standalone, quantitatively exact thermochemical predictions, which is consistent with the consensus philosophy of this study (Section 4.8).

For molecular docking, the ligand preparation procedure, protein preparation procedure, docking region, and scoring approach were kept consistent across the compound set. Docking energies were recorded for each compound, and the same conversion rule was applied to generate docking-derived indices. The docking-derived indices were then integrated with similarity-based indices using the predefined consensus procedure [55].

All the categorical classifications were assigned according to predefined rules. Uncertain or partially contradictory results were marked with a question mark to distinguish them from fully concordant predictions. This approach was used to avoid overinterpretation of inconsistent computational outputs.

To support reproducibility, the Supplementary Materials include the structural classification of the compounds, the computational workflow summary, the key numerical outputs used for consensus classification, the final consensus categories, and the modeled oxidation products used in the quantum-chemical antioxidant assessment.

### 4.10. In Silico ADMET Prediction

The pharmacokinetic and toxicity profiles of the twelve selected isoflavones (daidzin, genistin, acetyldaidzin, malonylgenistin, glycitin, acetylglycitin, biochanin A, formononetin, genistein, glycitein, puerarin, and daidzein) were predicted in silico using the Deep-PK web server (https://biosig.lab.uq.edu.au/deeppk/ (accessed on 23 July 2026)), a deep-learning-based platform for pharmacokinetic and toxicity property prediction, analysis, and optimization [18]. Deep-PK employs directed message-passing graph neural networks (D-MPNN, Chemprop framework) combined with 216 graph-based structural signatures, 37 toxicophore counts, and 196 RDKit molecular descriptors as additional graph-level features and provides predictions for 73 pharmacokinetic and toxicity endpoints together with 9 general physicochemical properties, covering the absorption, distribution, metabolism, excretion, and toxicity (ADMET) domains.

Two-dimensional chemical structures of the twelve isoflavones were retrieved from PubChem, and the corresponding canonical SMILES (Simplified Molecular Input Line Entry System) strings were used as inputs. Each SMILES string was individually submitted to the Deep-PK prediction module, which internally performs molecular sanitization and canonicalization using RDKit prior to model inference, as previously described for the underlying platform [18]. All available endpoints relevant to the four core pharmacokinetic domains and to toxicity were selected for prediction, including (i) absorption—Caco-2 permeability, human intestinal absorption, human oral bioavailability (20% and 50% thresholds), P-glycoprotein inhibition and substrate status; (ii) distribution—blood–brain barrier permeability/CNS penetration, plasma protein binding, fraction unbound (human), and steady-state volume of distribution; (iii) metabolism—inhibition/substrate status for the major cytochrome P450 isoforms (CYP1A2, CYP2C9, CYP2C19, CYP2D6, CYP3A4), breast cancer resistance protein (BCRP) inhibition, and organic anion-transporting polypeptide (OATP1B1/OATP1B3) inhibition; (iv) excretion—total clearance and elimination half-life; and (v) toxicity—AMES mutagenicity, carcinogenic potential, and drug-induced liver injury (DILI-I and DILI-II). Numerical (regression) outputs are reported using the model-specific units defined for each endpoint (e.g., log Papp in cm/s for Caco-2 permeability, mL/min/kg for total clearance), while categorical (classification) outputs are reported as the predicted class (e.g., inhibitor/noninhibitor, bioavailable/nonbioavailable), following the reference ranges and interpretive cut-offs established for the Deep-PK models (Deep-PK Theory documentation, https://biosig.lab.uq.edu.au/deeppk/theory (accessed on 23 July 2026)).

For each prediction, Deep-PK additionally reports a confidence tag (low, medium, or high) derived from whether the molecular weight of the query molecule and, for regression endpoints, its predicted value, which falls within the 95% distribution range of the corresponding training dataset [18]. This confidence metric was retained alongside each predicted value/class and is reported throughout the Section 2.8, as it reflects the degree to which a given isoflavone lies within the applicability domain of the respective model rather than the statistical probability of the prediction itself. All predictions were generated using the default deep-PK models (a single 3-fold cross-validated ensemble per endpoint) without further optimization or retraining, and the results were exported in tabular (CSV) format directly from the web server for compilation into the summary tables presented below (Table 8, Table 9, Table 10, Table 11 and Table 12).

Because deep-PK outputs represent machine-learning-based extrapolations from curated experimental training data rather than direct experimental measurements [18], all predicted ADMET parameters were interpreted conservatively, with particular caution applied to endpoints associated with low or medium confidence scores, and are intended to guide subsequent experimental validation rather than to substitute for it.

## 5. Conclusions

This study presents a consensus in silico pharmacodynamic evaluation of twelve representative Fabaceae isoflavones across five mechanistically relevant domains associated with type 2 diabetes mellitus: antiglycation, deglycation, antidiabetic, antioxidant, and NF-κB-related anti-inflammatory activity. Rather than identifying a single universally superior compound, the consensus analysis demonstrated that different isoflavones were prioritized for different pharmacodynamic endpoints. Malonylgenistin, biochanin A, and formononetin showed the most favorable predicted antiglycating profiles; genistin, acetyldaidzin, acetylglycitin, and puerarin were prioritized for predicted antidiabetic activity; genistein showed the most consistent predicted antioxidant profile, albeit with reduced confidence due to partial disagreement between the similarity-based and quantum-chemical methods; and daidzin together with genistin achieved the highest integrated NF-κB-related computational rankings, although it should be noted that predictions for the O-glycosidic candidates (genistin, acetyldaidzin, daidzin) reflect the intact glycoside rather than the aglycone species expected to predominate systemically following intestinal hydrolysis.

The combined use of complementary computational approaches, including IT Microcosm, PASS, quantum-chemical antioxidant modeling, and molecular docking exclusively for the NF-κB-related anti-inflammatory analysis, provided a comparative prioritization framework that reduces the reliance on a single predictive method and facilitates the selection of candidates for subsequent investigation. The results also emphasize that structural features, particularly glycosylation and acylation, substantially influence the predicted pharmacodynamic profiles of Fabaceae isoflavones.

Taken together, these findings should be interpreted strictly as comparative in silico prioritization rather than evidence of biological activity. The reported activities are computational predictions only and require confirmation through biochemical, cellular, pharmacokinetic (including ADMET), and in vivo studies before any therapeutic potential can be claimed.

## Figures and Tables

**Table 1:** Predicted antiglycating activity of the selected isoflavones using information technology Microcosm consensus strategies.

Compound	Conservative Strategy	Normal Strategy	Risk-Based Strategy	% Agreement	Final Consensus
Daidzein	Moderate	—	—	33%	Moderate?
Glycitein	Moderate	Moderate	Moderate	100%	Moderate
Genistein	—	—	Moderate	33%	Moderate?
Puerarin	Moderate	—	—	33%	Moderate?
Daidzin	Moderate	Moderate	Moderate	100%	Moderate
Acetyldaidzin	Moderate	Moderate	Moderate	100%	Moderate
Glycitin	Moderate	Moderate	Moderate	100%	Moderate
Acetylglycitin	Moderate	Moderate	Moderate	100%	Moderate
Genistin	Moderate	Moderate	Moderate	100%	Moderate
Malonylgenistin	Moderate	Moderate	High	33%	High?
Biochanin A	High	—	—	33%	High?
Formononetin	High	—	—	33%	High?

“—” indicates contradictory or nonconclusive prediction outputs. The additional “?” indicates reduced confidence in the assigned activity level because of partial divergence or incomplete concordance among computational outputs.

**Table 2:** Predicted deglycating activity of the selected isoflavones using information technology Microcosm consensus strategies.

Compound	Conservative Strategy	Normal Strategy	Risk-Based Strategy	% Agreement	Final Consensus
Acetyldaidzin	Moderate	Moderate	Moderate	100%	Moderate
Acetylglycitin	Moderate	Moderate	Moderate	100%	Moderate
Biochanin A	Moderate	Moderate	Moderate	100%	Moderate
Daidzein	Moderate	Moderate	Low	67%	Moderate?
Daidzin	Moderate	Moderate	Low	67%	Moderate?
Formononetin	Moderate	Moderate	Moderate	100%	Moderate
Glycitin	Moderate	Moderate	Moderate	100%	Moderate
Malonylgenistin	Moderate	Moderate	Moderate	100%	Moderate
Genistein	Moderate	Moderate	—	67%	Moderate?
Genistin	Moderate	Moderate	Low	67%	Moderate?
Puerarin	Moderate	Moderate	Low	67%	Moderate?
Glycitein	Low	Moderate	Low	67%	Low

“—” indicates contradictory or nonconclusive prediction output. The additional “?” indicates reduced confidence in the assigned activity level because of partial divergence or incomplete concordance among computational outputs.

**Table 3:** Prediction of Activity Spectra for Substances (PASS)-based prediction of the hypoglycemic and antidiabetic activity of the selected isoflavones.

Compound	Hypoglycemic Pa/Pi	Hypoglycemic Ind	Antidiabetic Pa/Pi	Antidiabetic Ind	PASS Consensus Pa/Pi	PASS Consensus Ind
Puerarin	106.60	3	150.60	3	128.60	3.0
Genistin	163.50	3	71.56	2	117.53	2.5
Daidzin	147.75	3	71.11	2	109.43	2.5
Acetyldaidzin	161.00	3	27.89	2	94.45	2.5
Acetylglycitin	113.20	3	11.97	2	62.59	2.5
Glycitin	84.00	2	30.00	2	57.00	2.0
Malonylgenistin	59.63	2	32.47	2	46.05	2.0
Biochanin A	17.43	2	0.51	0	8.97	1.0
Genistein	15.57	2	0.55	0	8.06	1.0
Formononetin	8.58	1	0.28	0	4.43	0.5
Daidzein	7.69	1	0.27	0	3.98	0.5
Glycitein	6.93	1	0.13	0	3.53	0.5

**Table 4:** Final consensus prediction of the hypoglycemic/antidiabetic activity of the selected isoflavones.

Compound	TMax	IndM	PASS Pa/Pi	IndP	IndC	Final Level
Acetylglycitin	0.565	2.1	62.6	2.5	2.3	High
Puerarin	0.350	1.1	128.6	3.0	2.0	High
Genistin	0.531	1.5	117.5	2.5	2.0	High
Acetyldaidzin	0.736	1.3	94.4	2.5	1.9	High
Daidzin	0.434	0.5	109.4	2.5	1.5	Moderate
Malonylgenistin	0.599	0.9	46.0	2.0	1.5	Moderate
Biochanin A	0.566	1.3	9.0	1.0	1.1	Moderate
Glycitin	0.541	0.3	57.0	2.0	1.1	Moderate
Formononetin	0.611	1.7	4.4	0.5	1.1	Moderate
Daidzein	0.433	1.1	4.0	0.5	0.8	Moderate
Genistein	0.411	0.3	8.1	1.0	0.6	Low
Glycitein	0.561	0.5	3.5	0.5	0.5	Low

TMax is the mean maximum QL-modified Tanimoto similarity coefficient to reference compounds in the information technology (IT) Microcosm. IndM is the IT Microcosm-derived activity index. IndP is the PASS-derived activity index. IndC is the integrated consensus index.

**Table 5:** Integrated antioxidant prediction of the selected isoflavones based on similarity-based and quantum-chemical approaches.

Compound	Similarity-Based Prediction	TQL	ΔGMin	Quantum-Chemical Classification	% Agreement	Final Antioxidant Consensus
Daidzein	Moderate	0.8917	−26.58	Above moderate	50%	High?
Glycitein	Moderate	1.0000	−28.70	Above moderate	50%	High?
Genistein	High	0.8218	−27.16	Moderate	50%	High?
Puerarin	Moderate	0.6103	−34.61	Above moderate	50%	High?
Daidzin	Moderate	0.9702	−36.06	Below moderate	50%	Low?
Acetyldaidzin	Low	0.7432	−38.95	Above moderate	50%	Moderate?
Glycitin	Low	0.9577	−36.92	Below moderate	100%	Low
Acetylglycitin	Low	0.8093	−39.75	Moderate	50%	Low?
Genistin	Moderate	0.9721	−35.21	Moderate	100%	Moderate
Malonylgenistin	Low	0.6818	−41.27	Above moderate	50%	Moderate?
Biochanin A	Moderate	0.9675	−28.09	Below moderate	50%	Low?
Formononetin	Moderate	0.9649	−27.74	Above moderate	50%	High?

TQL is the maximum similarity coefficient of QL-modified Tanimoto compounds to antioxidant reference compounds. ΔGMin is the lowest Gibbs free energy value among the modeled hydroxyl radical oxidation pathways; ΔGmin values were computed at the PM7 semiempirical level without explicit solvent correction and are intended for relative comparison only. The additional “?” indicates reduced confidence in the assigned activity level because of partial divergence or incomplete concordance among computational outputs.

**Table 6:** Integrated NF-κB-related computational ranking based on similarity and molecular docking.

Compound	IndMC_BioS	Docking Energy ΔE, kcal/mol	IndDock	IndNF-κB	Final Level
Genistin	0.0	−9.4	4.1	2.1	High
Daidzin	−0.1	−9.4	4.1	2.0	High
Acetyldaidzin	−0.7	−9.0	3.6	1.4	Moderate
Glycitin	0.0	−8.2	2.4	1.2	Moderate
Formononetin	0.1	−8.1	2.3	1.2	Moderate
Malonylgenistin	−0.8	−8.7	3.1	1.2	Moderate
Biochanin A	0.0	−8.1	2.3	1.1	Moderate
Daidzein	0.1	−7.8	1.9	1.0	Moderate
Puerarin	−0.3	−8.1	2.3	1.0	Moderate
Genistein	−0.7	−8.1	2.3	0.8	Moderate
Glycitein	−1.1	−8.1	2.3	0.6	Moderate
Acetylglycitin	−1.3	−8.1	2.3	0.5	Moderate

**Table 7:** Integrated multifunctional consensus profiles of the selected isoflavones.

Compound	Antiglycating Activity	Deglycating Activity	Antidiabetic Activity	Antioxidant Activity	NF-κB-Related Activity	Overall Consensus
Daidzin	Moderate?	Moderate?	Moderate	Low?	High	Moderate
Genistin	Moderate	Moderate?	High	Moderate	High	Moderate
Acetyldaidzin	Moderate	Moderate	High	Moderate?	Moderate	Moderate
Malonylgenistin	High?	Moderate	Moderate	Moderate?	Moderate	Moderate
Glycitin	Moderate	Moderate	Moderate	Low	Moderate	Moderate
Acetylglycitin	Moderate	Moderate	High	Low?	Moderate	Moderate
Biochanin A	High?	Moderate	Moderate	Low?	Moderate	Moderate
Formononetin	High?	Moderate	Moderate	High?	Moderate	Moderate
Genistein	Moderate?	Moderate?	Low	High?	Moderate	Moderate
Glycitein	Moderate	Low	Low	High?	Moderate	Moderate
Puerarin	Moderate?	Moderate?	High	High?	Moderate	Moderate
Daidzein	Moderate	Moderate?	Moderate	High?	Moderate	Moderate

The question mark indicates reduced confidence in the assigned activity category because of partial divergence between computational outputs. Consensus was assigned on the basis of the integrated profile across all five activity domains and was used for comparative prioritization rather than confirmation of biological efficacy.

**Table 8:** Absorption properties of selected isoflavones based on absorption, distribution, metabolism, excretion and toxicity in silico screening.

Compound	Caco-2 (log Paap)	Human Oral Bioavailability 20%	Human Intestinal Absorption	Human Oral Bioavailability 50%	P-Glycoprotein Inhibitor	P-Glycoprotein Substrate
Daidzin	−6.34	Bioavailable (Low Confidence)	Absorbed (Medium Confidence)	Non-Bioavailable (Low Confidence)	Non-Inhibitor (High Confidence)	Non-Substrate (Low Confidence)
Genistin	−6.64	Non-Bioavailable (Low Confidence)	Absorbed (Low Confidence)	Non-Bioavailable (Medium Confidence)	Non-Inhibitor (High Confidence)	Non-Substrate (Low Confidence)
Acetyldaidzin	−6.63	Non-Bioavailable (Low Confidence)	Absorbed (Low Confidence)	Non-Bioavailable (Low Confidence)	Non-Inhibitor (Low Confidence)	Non-Substrate (Low Confidence)
Malonylgenistin	−6.99	Non-Bioavailable (Medium Confidence)	Non-Absorbed (Medium Confidence)	Non-Bioavailable (Low Confidence)	Non-Inhibitor (High Confidence)	Substrate (Low Confidence)
Glycitin	−6.54	Non-Bioavailable (Low Confidence)	Absorbed (Low Confidence)	Non-Bioavailable (Medium Confidence)	Non-Inhibitor (High Confidence)	Non-Substrate (Low Confidence)
Acetylglycitin	−6.60	Non-Bioavailable (Low Confidence)	Absorbed (Low Confidence)	Non-Bioavailable (Medium Confidence)	Non-Inhibitor (High Confidence)	Substrate (Low Confidence)
Biochanin A	−4.77	Bioavailable (Medium Confidence)	Absorbed (High Confidence)	Bioavailable (Low Confidence)	Non-Inhibitor (High Confidence)	Non-Substrate (High Confidence)
Formononetin	−4.84	Bioavailable (Medium Confidence)	Absorbed (High Confidence)	Bioavailable (Low Confidence)	Inhibitor (High Confidence)	Non-Substrate (High Confidence)
Genistein	−4.94	Bioavailable (Low Confidence)	Absorbed (High Confidence)	Bioavailable (Low Confidence)	Non-Inhibitor (High Confidence)	Non-Substrate (High Confidence)
Glycitein	−4.69	Bioavailable (Low Confidence)	Absorbed (High Confidence)	Bioavailable (Low Confidence)	Inhibitor (High Confidence)	Non-Substrate (High Confidence)
Puerarin	−6.44	Non-Bioavailable (Low Confidence)	Non-Absorbed (Low Confidence)	Non-Bioavailable (Medium Confidence)	Non-Inhibitor (High Confidence)	Non-Substrate (Low Confidence)
Daidzein	−4.89	Bioavailable (Medium Confidence)	Absorbed (High Confidence)	Bioavailable (Low Confidence)	Non-Inhibitor (High Confidence)	Non-Substrate (High Confidence)

**Table 9:** Distribution properties of selected isoflavones based on absorption, distribution, metabolism, excretion and toxicity in silico screening.

Compound	CNS Permeability (log PS)	Predicted BBB Classification	Fraction-Unbound Model Output (Deep-PK Native Scale)	Plasma Protein Binding (%)	Steady State Volume of Distribution (L/kg)
Daidzin	−3.46	Non-penetrable (High Confidence)	1.13	91.84	0.86
Genistin	−3.72	Non-penetrable (High Confidence)	0.97	86.74	0.90
Acetyldaidzin	−3.75	Penetrable (High Confidence)	1.25	92.29	0.89
Malonylgenistin	−4.08	Non-penetrable (High Confidence)	1.10	80.26	0.93
Glycitin	−4.09	Non-penetrable (High Confidence)	1.17	91.47	0.77
Acetylglycitin	−4.06	Non-penetrable (Medium Confidence)	1.19	91.28	0.83
Biochanin A	−2.24	Penetrable (Medium Confidence)	1.20	81.33	0.36
Formononetin	−1.85	Penetrable (Low Confidence)	1.27	77.81	0.37
Genistein	−1.94	Non-penetrable (Medium Confidence)	0.82	80.28	0.34
Glycitein	−2.47	Non-penetrable (High Confidence)	1.40	81.27	0.30
Puerarin	−3.63	Non-penetrable (High Confidence)	0.92	87.25	0.91
Daidzein	−1.63	Non-penetrable (High Confidence)	0.91	74.64	0.48

Blood–Brain Barrier values are reported as log PS (log units); Fraction Unbound (Human) is reported on the Deep-PK model’s native regression scale and does not represent the literal 0–1 bound fraction-unbound value of classical pharmacokinetics; Plasma Protein Binding is expressed as percent bound (%); Steady-State Volume of Distribution is expressed in L/kg [18].

**Table 10:** In silico screening of the metabolic properties of selected isoflavones.

Compound	BCRP	CYP 1A2		CYP 2C19		CYP 2C9		CYP 2D6		CYP 3A4		OATP1B1	OATP1B3
		Inh.	Sub.	Inh.	Sub.	Inh.	Sub.	Inh.	Sub.	Inh.	Sub.		
Daidzin	−	−	−	−	−	−	+	−	−	−	−	−	−
Genistin	−	−	−	−	−	−	−	−	−	−	−	−	−
Acetyldaidzin	−	−	−	−	−	−	+	−	−	−	−	−	−
Malonylgenistin	+	−	−	−	−	−	+	−	−	−	−	−	−
Glycitin	−	−	−	−	−	−	+	−	−	−	−	−	−
Acetylglycitin	+	−	−	−	−	−	+	−	−	−	−	−	−
Biochanin A	+	+	−	+	−	−	+	+	−	+	−	−	−
Formononetin	+	+	+	+	−	−	+	−	+	+	−	−	−
Genistein	+	+	−	+	−	−	+	−	−	+	−	−	−
Glycitein	+	+	−	+	−	−	+	+	−	+	−	−	−
Puerarin	−	−	−	−	−	−	+	−	−	−	−	−	−
Daidzein	+	+	−	+	−	−	+	−	+	+	−	−	−

Note: BCRP—Breast Cancer Resistance Protein; Inh.—inhibitor; Sub.—substrate; + indicates predicted inhibitor/substrate status; − indicates predicted non-inhibitor/non-substrate status, according to the corresponding column.

**Table 11:** Excretion properties of selected isoflavones based on ADMET in silico screening.

Compound	Clearance	Half-Life of Drug
Daidzin	8.26	Half-Life < 3 h (Low Confidence)
Genistin	11.18	Half-Life < 3 h (Medium Confidence)
Acetyldaidzin	7.73	Half-Life < 3 h (Low Confidence)
Malonylgenistin	7.30	Half-Life < 3 h (Low Confidence)
Glycitin	7.65	Half-Life < 3 h (Low Confidence)
Acetylglycitin	7.34	Half-Life < 3 h (Low Confidence)
Biochanin A	3.04	Half-Life < 3 h (Medium Confidence)
Formononetin	4.55	Half-Life < 3 h (Medium Confidence)
Genistein	3.27	Half-Life < 3 h (Medium Confidence)
Glycitein	3.31	Half-Life < 3 h (Medium Confidence)
Puerarin	9.59	Half-Life < 3 h (Low Confidence)
Daidzein	3.17	Half-Life < 3 h (Medium Confidence)

Clearance is expressed as total predicted clearance in mL/min/kg body weight (composite, non-organ-specific estimate). The half-life of drugs is reported by Deep-PK as a categorical classification (<3 h vs. ≥3 h) rather than a continuous numerical value; the accompanying confidence tag (low/medium/high) reflects whether the query compound falls within the applicability domain of the underlying classification model, not a statistical probability of the class assignment [18].

**Table 12:** Toxicity properties of selected isoflavones based on ADMET in silico screening.

Compound	AMES Mutagenesis	Carcinogenesis	Liver Injury I	Liver Injury II
Daidzin	Toxic (High Confidence)	Safe (High Confidence)	Safe (Low Confidence)	Toxic (Medium Confidence)
Genistin	Toxic (High Confidence)	Safe (High Confidence)	Safe (Low Confidence)	Toxic (Medium Confidence)
Acetyldaidzin	Toxic (High Confidence)	Safe (High Confidence)	Safe (Medium Confidence)	Toxic (Medium Confidence)
Malonylgenistin	Toxic (High Confidence)	Safe (High Confidence)	Safe (Medium Confidence)	Toxic (Medium Confidence)
Glycitin	Toxic (High Confidence)	Safe (High Confidence)	Safe (Medium Confidence)	Toxic (Medium Confidence)
Acetylglycitin	Toxic (High Confidence)	Safe (High Confidence)	Safe (Medium Confidence)	Toxic (Medium Confidence)
Biochanin A	Safe (High Confidence)	Safe (Low Confidence)	Toxic (Medium Confidence)	Toxic (Medium Confidence)
Formononetin	Safe (Medium Confidence)	Safe (Low Confidence)	Toxic (Low Confidence)	Toxic (Medium Confidence)
Genistein	Safe (High Confidence)	Toxic (Low Confidence)	Toxic (Medium Confidence)	Toxic (Low Confidence)
Glycitein	Safe (High Confidence)	Safe (Medium Confidence)	Toxic (Medium Confidence)	Toxic (Medium Confidence)
Puerarin	Toxic (High Confidence)	Safe (High Confidence)	Safe (Low Confidence)	Toxic (Medium Confidence)
Daidzein	Safe (High Confidence)	Safe (Low Confidence)	Toxic (Medium Confidence)	Toxic (Medium Confidence)

**Table 13:** Modeled hydroxyl radical oxidation pathways used for quantum-chemical antioxidant evaluation of the selected isoflavones.

Oxidation Pathway	Isoflavones Included in the Pathway
Pathway 1	Acetyldaidzin, acetylglycitin, daidzein, daidzin, genistein, genistin, glycitein, glycitin, malonylgenistin, puerarin
Pathway 2	Biochanin A, daidzein, formononetin, genistein, genistin, glycitein, malonylgenistin, puerarin

## Data Availability

The original contributions presented in this study are included in the article/supplementary material. Further inquiries can be directed to the corresponding authors.

## Supplementary Materials

The following supporting information can be downloaded at: https://www.mdpi.com/article/10.3390/ph19091353/s1, Table S1: Key numerical outputs used for consensus classification of the twelve isoflavones; Table S2: Structural information, Fabaceae occurrence, and rationale for inclusion of the twelve selected Fabaceae isoflavones used for in silico screening; Table S3: Computational methods, software, input data, and outputs used in the consensus in silico screening workflow; Figure S1: Proposed oxidation products generated for hydroxyl-radical reaction modeling of the selected Fabaceae isoflavones; Table S4: Three-dimensional structural models of NF-κB proteins and docking-grid parameters used for ensemble molecular docking.

## References

[1] Chen Y., Meng Z., Li Y., Liu S., Hu P., Luo E. (2024). Advanced glycation end products and reactive oxygen species: Uncovering the potential role of ferroptosis in diabetic complications. Mol. Med..

[2] Weinberg Sibony R., Segev O., Dor S., Raz I. (2024). Overview of oxidative stress and inflammation in diabetes. J. Diabetes.

[3] American Diabetes Association Professional Practice Committee (2024). 2. Diagnosis and Classification of Diabetes: Standards of Care in Diabetes—2025. Diabetes Care.

[4] Bennici G., Almahasheer H., Alghrably M., Valensin D., Kola A., Kokotidou C., Lachowicz J., Jaremko M. (2024). Mitigating diabetes associated with reactive oxygen species (ROS) and protein aggregation through pharmacological interventions. RSC Adv..

[5] Nxumalo M.B., Kumalo H.M., Maruma L., Tata F.Y., Ntanzi N., Khan R.B. (2026). Oxidative stress, inflammation, and metabolic dysregulation in type 2 diabetes: Therapeutic potential of Carica papaya. Health Sci. Rev..

[6] Zhao L., Yuan J., Yang Q., Ma J., Yang F., Zou Y., Liu K., Liu F. (2026). Diabetes and its complications: Molecular mechanisms, prevention and treatment. Signal Transduct. Target. Ther..

[7] Kuryłowicz A. (2021). The Role of Isoflavones in Type 2 Diabetes Prevention and Treatment—A Narrative Review. Int. J. Mol. Sci..

[8] Kamiyama M., Iijima K., Okuzawa R., Kawata R., Kimura A., Shinohara Y., Shimada A., Yamanaka M., Youda A., Iwamoto T. (2025). Mechanisms of the Effects of Polyphenols on Diabetic Nephropathy. Curr. Issues Mol. Biol..

[9] Kciuk M., Kruczkowska W., Wanke K., Gałęziewska J., Kołat D., Mujwar S., Kontek R. (2025). The Role of Genistein in Type 2 Diabetes and Beyond: Mechanisms and Therapeutic Potential. Molecules.

[10] Camacho-Padilla L.G., Zepeda-Morales A.S.M., Arrizon J., Flores-Soto M.E., Zepeda-Nuño J.S., Carrera-Quintanar L., Herrera-González A., Aparicio-García G., López-Roa R.I. (2025). Preclinical Evaluation of Puerarin as a Modulator of Metabolic and Inflammatory Parameters in a High-Fat-Diet-Fed Mice Model. Molecules.

[11] Mahajan U.B., Goyal S. (2025). Biochanin A Mitigates Oxidative Stress and Inflammation in Diabetic Myocardial Infarction: Insights from a Streptozotocin and Isoproterenol Rat Model. Cureus.

[12] Ma P., Gao Y., Wang H., Jiang X., Tang Y., Gong M., Gong J., Dong H., Su H. (2026). The therapeutic potential of puerarin on diabetic kidney disease: Focus on molecular mechanisms and future perspectives. Pharmacol. Res..

[13] Liga S., Paul C. (2024). Puerarin—A Promising Flavonoid: Biosynthesis, Extraction Methods, Analytical Techniques, and Biological Effects. Int. J. Mol. Sci..

[14] Jin Q., Liu T., Qiao Y., Liu D., Yang L., Mao H., Ma F., Wang Y., Peng L., Zhan Y. (2023). Oxidative stress and inflammation in diabetic nephropathy: Role of polyphenols. Front. Immunol..

[15] de Angelo R.M., de Sousa D.S., da Silva A.P., Chiari L.P.A., da Silva A.B.F., Honorio K.M. (2024). Molecular Docking: State-of-the-Art Scoring Functions and Search Algorithms. Computer-Aided and Machine Learning-Driven Drug Design: From Theory to Applications.

[16] Langa S., Peirotén Á., Curiel J.A., de la Bastida A.R., Landete J.M. (2023). Isoflavone Metabolism by Lactic Acid Bacteria and Its Application in the Development of Fermented Soy Food with Beneficial Effects on Human Health. Foods.

[17] Bai Y.-L., Han L.-L., Qian J.-H., Wang H.-Z. (2022). Molecular Mechanism of Puerarin Against Diabetes and its Complications. Front. Pharmacol..

[18] Myung Y., de Sá A.G.C., Ascher D.B. (2024). Deep-PK: Deep learning for small molecule pharmacokinetic and toxicity prediction. Nucleic Acids Res..

[19] Jing X., Zhou J., Zhang N., Zhao L., Wang S., Zhang L., Zhou F. (2022). A Review of the Effects of Puerarin on Glucose and Lipid Metabolism in Metabolic Syndrome: Mechanisms and Opportunities. Foods.

[20] Jain R., Bolch C., Al-Nakkash L., Sweazea K.L. (2022). Systematic review of the impact of genistein on diabetes-related outcomes. Am. J. Physiol.-Regul. Integr. Comp. Physiol..

[21] Goleij P., Sanaye P.M., Alam W., Zhang J., Tabari M.A.K., Filosa R., Jeandet P., Cheang W.S., Efferth T., Khan H. (2024). Unlocking daidzein’s healing power: Present applications and future possibilities in phytomedicine. Phytomedicine.

[22] Zhuo J.-J., Li B.-J., Tian M.-R. (2026). Biological Activities, Health Benefits and Synthesis of Equol and Its Derivatives: A Comprehensive Review. Food Sci. Nutr..

[23] Fan H., Yang J., Wang H., Zong S., Qu Y., Wang K., Zhang F., Li X. (2025). Efficacy and safety of puerarin injection as an adjunctive therapy for chronic heart failure: A systematic review and meta-analysis. Front. Pharmacol..

[24] Goh Y.X., Jalil J., Lam K.W., Husain K., Premakumar C.M. (2022). Genistein: A Review on its Anti-Inflammatory Properties. Front. Pharmacol..

[25] Mengstie M.A., Chekol Abebe E., Behaile Teklemariam A., Tilahun Mulu A., Agidew M.M., Teshome Azezew M., Zewde E.A., Agegnehu Teshome A. (2022). Endogenous advanced glycation end products in the pathogenesis of chronic diabetic complications. Front. Mol. Biosci..

[26] Lee J., Yun J.-S., Ko S.-H. (2022). Advanced Glycation End Products and Their Effect on Vascular Complications in Type 2 Diabetes Mellitus. Nutrients.

[27] Spagnuolo L., Della Posta S., Fanali C., Dugo L., De Gara L. (2021). Antioxidant and Antiglycation Effects of Polyphenol Compounds Extracted from Hazelnut Skin on Advanced Glycation End-Products (AGEs) Formation. Antioxidants.

[28] Maisto M., Tenore G.C. (2024). Polyphenols as a Useful Tool to Ameliorate Advanced Glycation End-product Formation: A Focus on Molecular Mechanisms of Action. Front. Biosci. (Landmark Ed).

[29] Chociej P., Foss K., Jabłońska M., Ustarbowska M., Sawicki T. (2024). The Profile and Content of Polyphenolic Compounds and Antioxidant and Anti-Glycation Properties of Root Extracts of Selected Medicinal Herbs. Plant Foods Hum. Nutr..

[30] Agu P.C., Afiukwa C.A., Orji O.U., Ezeh E.M., Ofoke I.H., Ogbu C.O., Ugwuja E.I., Aja P.M. (2023). Molecular docking as a tool for the discovery of molecular targets of nutraceuticals in diseases management. Sci. Rep..

[31] Kim M.-S., Jung Y.S., Jang D., Cho C.H., Lee S.-H., Han N.S., Kim D.-O. (2022). Antioxidant capacity of 12 major soybean isoflavones and their bioavailability under simulated digestion and in human intestinal Caco-2 cells. Food Chem..

[32] Teodosio J.J.R., Dizon K.A.H., Bruna J.R., Sollesta J.V.N., Villorente Z.M., Saludes J.P., Dalisay D.S. (2026). Biochanin A, a Plant Isoflavone, Disrupts Peptidoglycan Biosynthesis by Downregulating femA and femB, and Impairs Cell Wall Integrity in Multidrug-Resistant Staphylococcus aureus. Antibiotics.

[33] Kwieciński M., Kwiatkowski B., Ziomek W., Bomba W., Wróblewska-Łuczka P. (2026). Biological activities and therapeutic potential of soy isoflavones: A focus on anticancer activity. Mol. Biol. Rep..

[34] Yue S., Ye X., Nan T., Li C., Huang L., Yuan Y. (2025). Daidzin from Pueraria thomsonii regulate root plasticity and cell proliferation. Ind. Crops Prod..

[35] Sharma N., Kabra A. (2025). Formononetin: Pharmacological properties and therapeutic potential. Naunyn-Schmiedeberg’s Arch. Pharmacol..

[36] Katanić Stanković J.S., Mihailović N., Mihailović V. (2023). Genistein: Advances on Resources, Biosynthesis Pathway, Bioavailability, Bioactivity, and Pharmacology. Handbook of Dietary Flavonoids.

[37] Lu F., Wang Y., Wu S., Huang W., Yao H., Wang S., Shi X., Laborda P., Herrera-Balandrano D.D. (2024). Germination time and in vitro gastrointestinal digestion impact on the isoflavone bioaccessibility and antioxidant capacities of soybean sprouts. Food Chem..

[38] Wu H., Feng Y., Xie X., Zhang Y. (2025). Glycitin in Soy Beans: A Mine of Their Structures, Functions, and Potential Applications. Foods.

[39] Yang Q., Wang G. (2024). Isoflavonoid metabolism in leguminous plants: An update and perspectives. Front. Plant Sci..

[40] Mitharwal S., Saini A., Chauhan K., Taneja N.K., Oberoi H.S. (2024). Unveiling the nutrient-wealth of black soybean: A holistic review of its bioactive compounds and health implications. Compr. Rev. Food Sci. Food Saf..

[41] Certara Chemaxon Marvin: Chemical Drawing Software. https://www.certara.com/marvin/.

[42] Vassiliev P.M., Spasov A.A., Kosolapov V.A., Kucheryavenko A.F., Gurova N.A., Anisimova V.A. (2014). Consensus Drug Design Using IT Microcosm. Application of Computational Techniques in Pharmacy and Medicine.

[43] Zdrazil B., Felix E., Hunter F., Manners E.J., Blackshaw J., Corbett S., de Veij M., Ioannidis H., Lopez D.M., Mosquera J.F. (2024). The ChEMBL Database in 2023: A drug discovery platform spanning multiple bioactivity data types and time periods. Nucleic Acids Res..

[44] Dao Q.T., Do T.M.D., Thai Q.M., Tran P.-T., Ngo S.T., Nguyen T.H. (2026). Identifying Potential BACE1 Inhibitors from the ChEMBL Database Using Machine Learning and Atomistic Simulation Approaches. ACS Omega.

[45] Filimonov D.A., Lagunin A.A., Gloriozova T.A., Rudik A.V., Druzhilovskii D.S., Pogodin P.V., Poroikov V.V. (2014). Prediction of the Biological Activity Spectra of Organic Compounds Using the Pass Online Web Resource. Chem. Heterocycl. Compd..

[46] Basar Y., Yenigun S., Yılmaz S., Kocaman A.Y., Demirtas I., Alma M.H., Temel S., Ozen T. (2025). Urease inhibitory potency of Chenopodium quinoa seed extract: LC–ESI–MS/MS, GC–MS/MS analysis, biological evaluations, molecular docking, ADMET property, DFT calculation and PASS prediction. J. Food Meas. Charact..

[47] Vesnina A., Le V., Ivanova S., Prosekov A. (2025). Antidiabetic Potential of Mangiferin: An In Silico and In Vivo Approach. Pharmaceutics.

[48] Cárdenas M.A., Alaníz R.D., Crapnell R.D., Robledo S.N., Fernández H., Arévalo F.J., Granero A.M., Banks C.E., Pierini G.D. (2025). Electrochemically Activated Screen-Printed Graphene Electrochemical Sensor for Daidzein Determination in Edible Peanut Oils. Chemosensors.

[49] Yang T., Qi F., Guo F., Shao M., Song Y., Ren G., Linlin Z., Qin G., Zhao Y. (2024). An update on chronic complications of diabetes mellitus: From molecular mechanisms to therapeutic strategies with a focus on metabolic memory. Mol. Med..

[50] Raval S.D., Archana G. (2024). Evaluation of synbiotic combinations of commercial probiotic strains with different prebiotics in in vitro and ex vivo human gut microcosm model. Arch. Microbiol..

[51] Boukerouis D., Cuadrado I., Benaida N.D., Estévez-Braun A., de las Heras B., Amesty A., Hortelano S. (2025). Exploring the anti-inflammatory activity of fupenzic acid using network pharmacology and experimental validation. Sci. Rep..

[52] Pasha A., Kumar K., Heena S.K., Arnold Emerson I., Pawar S.C. (2024). Inhibition of NF-kB and COX-2 by andrographolide regulates the progression of cervical cancer by promoting PTEN expression and suppressing PI3K/AKT signalling pathway. Sci. Rep..

[53] Zhou Y., Lin S., Zhong X., Huang F., Huang J., Xu L. (2024). Oleanolic acid combined with aspirin plays antitumor roles in colorectal cancer via the Akt/NFκB/IκBα/COX2 pathway. Cell Death Discov..

[54] Pagag J., Andola P., Durgam L., Guruprasad L. (2025). Computational design and validation of small molecule inhibitors for type III phosphatidylinositol-4-kinase alpha, a hepatitis C drug target. J. Biomol. Struct. Dyn..

[55] Martis E.A.F., Téletchéa S. (2025). Ten quick tips to perform meaningful and reproducible molecular docking calculations. PLoS Comput. Biol..

