# Supplementary material for: Consensus In Silico Pharmacodynamic Screening of Twelve Fabaceae Isoflavones Targeting Glycation, Oxidative Stress, Antidiabetic Activity, and NF-κB-Related Inflammation
Source: Pharmaceuticals (Basel). 2026 Aug 26;19(9):1353. doi: 10.3390/ph19091353 (PMC13610150; doi:10.3390/ph19091353)
Supplement: Supplementary file 1 [file pharmaceuticals-19-01353-s001.zip › pharmaceuticals-4389544-supplementary.pdf]

Article

Consensus In Silico Pharmacodynamic Screening of Twelve Fabaceae Isoflavones Targeting Glycation, Oxidative Stress, Antidiabetic Activity, and NF-κB-Related Inflammation

Saied A. Aboushanab [1,*], Denis A. Oberiukhtin [2], Irina G. Danilova [2,3], Pavel M. Vassiliev [4], Ksenia V. Sokolova [2,3], Kewei Chen [5], Elena G. Kovaleva [1], Alicia C. Mondragon [6], Ekaterina I. Demicheva [1,2] and Jose M. Miranda [6,*]

[1] Institute of Chemical Engineering, Ural Federal University Named After the First President of Russia B. N. Yeltsin, Mira 19, 620002 Yekaterinburg, Russia; e.g.kovaleva@urfu.ru (E.G.K.); fimby@mail.ru (E.I.D.)

[2] Institute of Immunology and Physiology, Ural Branch of the Russian Academy of Science, 106 Pervomaiskaya Street, 620049 Yekaterinburg, Russia; oberuhtindenis@gmail.com (D.A.O.); i.g.danilova@urfu.ru (I.G.D.); ksenia.sokolova@urfu.ru (K.V.S.)

[3] Institute of Natural Science and Mathematics, Ural Federal University Named After the First President of Russia B. N. Yeltsin, Mira 19, 620002 Yekaterinburg, Russia

[4] Department of Pharmacology and Bioinformatics, Volgograd State Medical University, 1 Pavshikh Bortsov Sq., 400131 Volgograd, Russia;
pvassiliev@mail.ru

[5] College of Food Science, Southwest University, No. 2, Tiansheng Road, Beibei, Chongqing 400715, China; chenkewei@swu.edu.cn

[6] Department of Analytical Chemistry, Nutrition and Food Science, Institute for Research in Global Health and Sustainable Development, iTERRA, School of Veterinary Sciences, University of Santiago de Compostela, 27002 Lugo, Spain; alicia.mondragon@usc.es

* Correspondence: sabushanab@urfu.ru (S.A.A.); josemanuel.miranda@usc.es (J.M.M.)

Table S1. Key numerical outputs used for consensus classification of the twelve isoflavones.

Compound	Antidiabetic IndC	Antioxidant TQL	Antioxidant ΔGMin	NF-κB IndMC_BioS	NF-κB IndDock	NF-κB IndNF-κB
Daidzein	0.8	0.8917	−26.58	0.1	1.9	1.0
Glycitein	0.5	1.0000	−28.70	−1.1	2.3	0.6
Genistein	0.6	0.8218	−27.16	−0.7	2.3	0.8
Puerarin	2.0	0.6103	−34.61	−0.3	2.3	1.0
Daidzin	1.5	0.9702	−36.06	−0.1	4.1	2.0
Acetyldaidzin	1.9	0.7432	−38.95	−0.7	3.6	1.4
Glycitin	1.1	0.9577	−36.92	0.0	2.4	1.2
Acetylglycitin	2.3	0.8093	−39.75	−1.3	2.3	0.5
Genistin	2.0	0.9721	−35.21	0.0	4.1	2.1
Malonylgenistin	1.5	0.6818	−41.27	−0.8	3.1	1.2
Biochanin A	1.1	0.9675	−28.09	0.0	2.3	1.1
Formononetin	1.1	0.9649	−27.74	0.1	2.3	1.2

IndC is the integrated consensus index for hypoglycemic/antidiabetic activity. TQL is the maximum QL-modified Tanimoto similarity coefficient to antioxidant reference compounds. ΔGMin is the selected Gibbs free energy value for the most favorable modeled hydroxyl radical oxidation pathway. IndMC_BioS is the Microcosm BioS similarity-derived NF-κB index. IndDock is the docking-derived NF-κB index. IndNF-κB is the integrated NF-κB consensus index.

Table S2. Structural information, Fabaceae occurrence, and rationale for inclusion of the twelve selected Fabaceae isoflavones used for in silico screening.

No.	Compound	Representative Fabaceae source/relevance	Molecular formula	Molecular weight, g/mol	Rationale for inclusion	Reference(s)
1	Daidzein	Soybean/*Pueraria*/*Trifolium* isoflavone	$C_{15}H_{10}O_4$	254.24	A representative nonmethoxylated aglycone used for comparison with glycosidic and methoxylated derivatives	[37,43]
2	Glycitein	Soybean-related isoflavone (*Glycine max*)	$C_{16}H_{12}O_5$	284.26	To evaluate the effect of methoxylation in the daidzein-related series	[37,42]
3	Genistein	Soybean/*Trifolium*/*Pueraria*	$C_{15}H_{10}O_5$	270.24	A representative hydroxylated aglycone with known antioxidant relevance	[43]
4	Puerarin	*Pueraria* species	$C_{21}H_{20}O_9$	416.38	A representative C-glycosidic isoflavone characteristic of *Pueraria* species	[42]
5	Daidzin	soybean/*Pueraria* materials	$C_{21}H_{20}O_9$	416.38	To compare O-glycosylation with aglycone and C-glycoside forms	[38]
6	Acetyldaidzin		$C_{23}H_{22}O_{10}$	458.42	To assess the possible contribution of acetylation to predicted activity	[35]
7	Glycitin	soybean-derived materials (*Glycine max*)	$C_{22}H_{22}O_{10}$	446.40	A methoxylated O-glycosidic derivative	[42]
8	Acetylglycitin		$C_{24}H_{24}O_{11}$	488.44	To compare acetylated glycosides within the glycitein-related series	[44]

9	Genistin		$C_{21}H_{20}O_{10}$	432.38	A main glycosidic derivative of genistein	[40]
10	Malonylgenistin		$C_{24}H_{22}O_{13}$	518.42	To assess the predicted activity of a malonylated glycosidic isoflavone	[41]
11	Biochanin A	*Trifolium pratense* and other Fabaceae species	$C_{16}H_{12}O_5$	284.26	To compare methoxylated and nonmethoxylated genistein-related aglycones	[36]
12	Formononetin		$C_{16}H_{12}O_4$	268.26	To compare methoxylated and nonmethoxylated daidzein-related aglycones	[39]

The molecular structures of the compounds were prepared and standardized before computational screening. The selected compounds represent major structural subclasses of Fabaceae isoflavones, including aglycones, C-glycosides, O-glycosides, acetylated O-glycosides, malonylated O-glycosides, and methoxylated aglycones. All twelve compounds were selected because they are isoflavone aglycones or naturally occurring isoflavone glycosides/acylated derivatives reported from Fabaceae-derived sources, including soybean-derived materials, *Pueraria* species, and *Trifolium pratense*.

Table S3. Computational methods, software, input data, and outputs used in the consensus in silico screening workflow.

Screening block	Software/platform	Input data	Main output	Role in consensus analysis
Molecular structure preparation	ChemOffice/ChemFinder; MarvinSketch	Two-dimensional structures of the twelve selected isoflavones	Standardized molecular structures in compatible chemical formats	Provided chemically consistent input structures for all subsequent prediction blocks
Antiglycating activity prediction	IT Microcosm, Testing module	Standardized molecular structures translated into QL descriptors	Predicted activity levels using conservative, normal, and risk-based strategies	Used to classify compounds as High, Moderate, Low, or non-conclusive for antiglycating potential
Deglycating activity prediction	IT Microcosm, Testing module	Standardized molecular structures translated into QL descriptors	Predicted activity levels using conservative, normal, and risk-based strategies	Used to classify compounds for predicted deglycating potential
Hypoglycemic activity prediction	IT Microcosm/TestSim	QL descriptor profiles of the selected isoflavones	TMax values and Microcosm-derived activity index	Provided similarity-based estimate of hypoglycemic potential
Hypoglycemic and antidiabetic prediction	PASS 10.4 Professional Extended	Standardized molecular structures	Pa, Pi, Pa/Pi ratio, and PASS-derived activity index	Provided probability-based prediction of hypoglycemic and antidiabetic activity
Antioxidant similarity-based prediction	IT Microcosm/TestSim	QL descriptor profiles of the selected isoflavones	TQL values and predicted antioxidant category	Provided similarity-based antioxidant classification
Quantum-chemical antioxidant modeling	MarvinSketch; MOPAC, PM7/THERMO	Parent isoflavones and modeled hydroxyl-radical oxidation products	Thermodynamic parameters and ΔG values for oxidation pathways	Provided thermodynamic support for antioxidant classification
NF-κB similarity-based prediction	Microcosm BioS	QL descriptor profiles of the selected isoflavones	TMean and IndMC_BioS values	Provided similarity-based prediction of NF-κB-related activity

NF-κB docking	PyRx 0.8; AutoDock Vina	Optimized 3D ligand structures and validated NF-κB-related protein models	Docking energy ΔE and docking-derived activity index	Provided binding-affinity component for NF-κB-related consensus classification
Final prioritization	Excel/manual consensus scoring	Outputs from all prediction blocks	Integrated activity-specific and multifunctional consensus profiles	Used to identify domain-specific and multifunctional priority compounds

QL descriptors were used in IT Microcosm/TestSim and Microcosm BioS workflows. PASS predictions were based on structural descriptors implemented in PASS. Docking-derived and similarity-derived NF-κB indices were integrated to generate the final NF-κB-related consensus score, whereas no docking-derived parameters were used in the hypoglycemic/antidiabetic prediction.

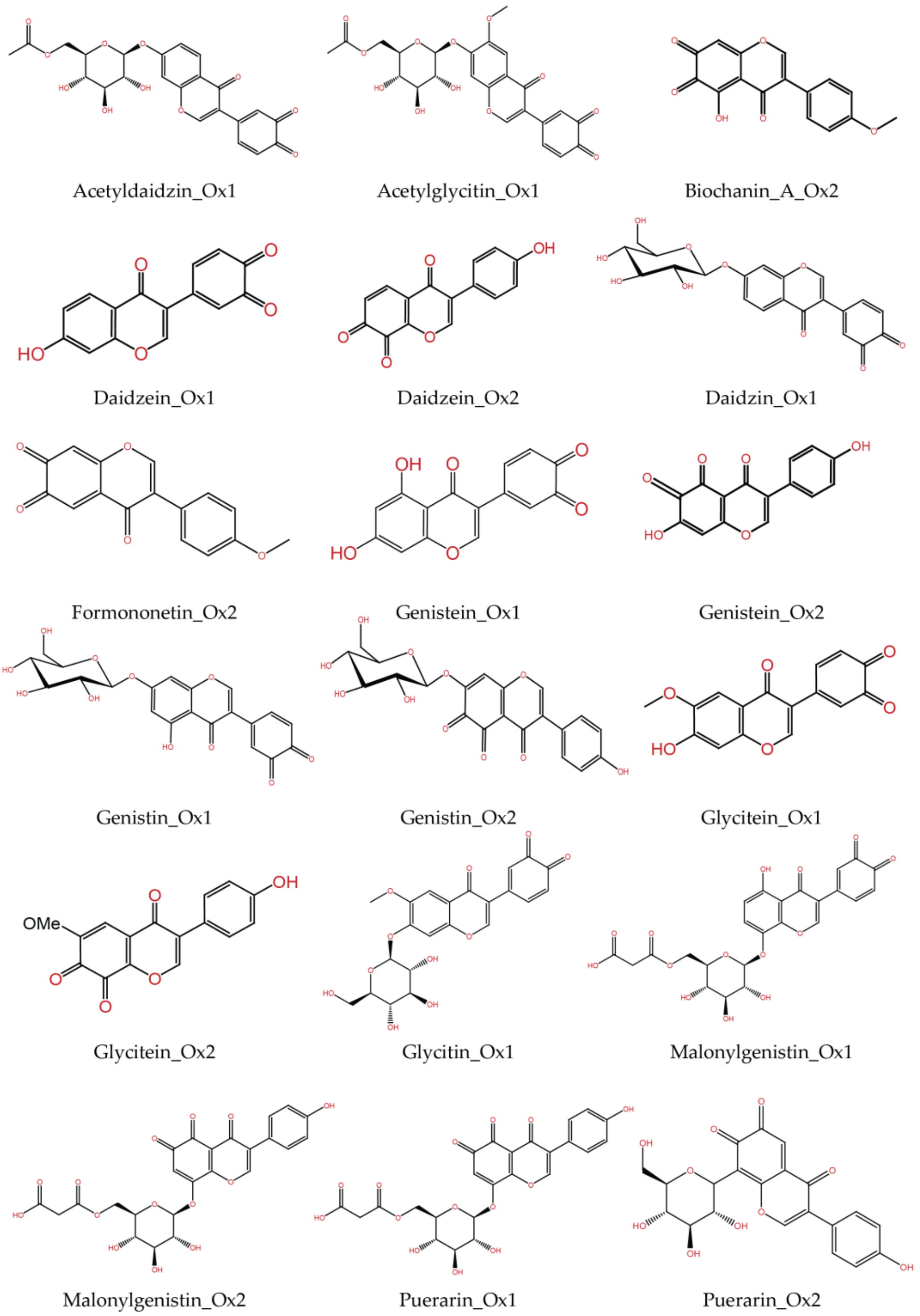

Figure S1. Proposed oxidation products generated for hydroxyl-radical reaction modeling of the selected Fabaceae isoflavones.

Products were modeled according to two probable oxidation pathways. Pathway 1 was applied to acetyldaidzin, acetylglycitin, daidzein, daidzin, genistein, genistin, glycitein, glycitin, malonylgenistin, and puerarin. Pathway 2 was applied to biochanin A, daidzein, formononetin, genistein, genistin, glycitein, malonylgenistin, and puerarin. These structures were used as oxidation products in the quantum-chemical calculation of Gibbs free energy changes.

Table S4. Three-dimensional structural models of NF-κB proteins and docking-grid parameters used for ensemble molecular docking.

NF-κB protein/subunit	Model identifier	Structure type	Source	Chain	Key residues	Grid center X	Grid center Y	Grid center Z	Grid size X, Å	Grid size Y, Å	Grid size Z, Å	Exhaustiveness	Number of poses
NFκB1 (p50)	2O61	Experimental	PDB	D [auth B]	Arg57, Cys62, Asn247	14.258	-6.417	22.903	24.0	24.0	24.0	8	9
NFκB1 (p50)	1SVC	Experimental	PDB	B [auth P]	Arg57, Cys62, Asn247	10.842	18.334	-4.215	24.0	24.0	24.0	8	9
NFκB1 (p50)	3GUT	Experimental	PDB	B	Arg57, Cys62, Asn247	-3.682	21.105	11.746	24.0	24.0	24.0	8	9
NFκB2 (p52)	534096906e83c5c6e7de519a2a83f8ad	Homology model	ModBase	—	Arg54, Thr142, Lys255	8.214	-11.635	17.528	24.0	24.0	24.0	8	9
NFκB2 (p52)	7f22a1f8f1402e08a160cd2f59c0a91a	Homology model	ModBase	—	Arg54, Thr142, Lys255	5.903	-8.472	20.116	24.0	24.0	24.0	8	9
NFκB2 (p52)	bb1cdc252d8b233e0fadbc070070ce46	Homology model	ModBase	—	Arg54, Thr142, Lys255	12.447	-14.206	9.774	24.0	24.0	24.0	8	9
RELA (p65)	1NFI	Experimental	PDB	A	Gln241, Arg187, Tyr188	12.458	-3.221	27.904	24.0	24.0	24.0	8	9
RELA (p65)	5926d2baf1091a72bfe859b160a46f5a	Homology model	ModBase	—	Gln241, Arg187, Tyr188	16.842	-2.518	25.663	24.0	24.0	24.0	8	9
RELA (p65)	d883c4ab1d9882778f5001c874316a19	Homology model	ModBase	—	Gln241, Arg187, Tyr188	11.276	-5.904	30.112	24.0	24.0	24.0	8	9

PDB, Protein Data Bank. The nine models used for ensemble molecular docking in the NF-κB-related anti-inflammatory analysis were selected from a previously evaluated pool of experimental and theoretical NF-κB structures. Experimental structures were obtained from the PDB, whereas theoretical models were obtained from ModBase. The table reports the available structural identifiers, selected protein chains where applicable, and the AutoDock Vina search-space parameters used for each model. Grid-center coordinates and grid dimensions are given in Å.
